# Sex Disparities in Infective Endocarditis Presentation, Management and Outcomes: A Systematic Review and Meta-Analysis

**DOI:** 10.3390/diagnostics16020260

**Published:** 2026-01-14

**Authors:** Hugh Jacobs, Arian Arjomandi Rad, Ahmad Walid Izzat, Gustavo Antonio Guida, Fadi Ibrahim Al-Zubaidi, Danilo Verdichizzo, Ihab Abu Reish, Rana Sayeed, Antonios Kourliouros

**Affiliations:** 1 Department of Cardiothoracic Surgery, Bristol Heart Institute, University Hospitals Bristol and Weston NHS Trust, Bristol BS2 8ED, UK; 2 Department of Cardiothoracic Surgery, Oxford Heart Centre, Oxford University Hospitals NHS Foundation Trust, Oxford OX3 9DU, UK

**Keywords:** infective endocarditis, sex differences, gender disparities, cardiac surgery, mortality, meta-analysis, systematic review

## Abstract

**Background:** Sex-based disparities in the presentation, management, and outcomes of infective endocarditis (IE) remain insufficiently characterized despite their growing recognition. This study systematically evaluates current evidence on sex differences in the presentation, treatment, and outcomes of IE. **Methods:** A systematic review and meta-analysis were conducted according to PRISMA and Cochrane guidelines. EMBASE, MEDLINE, PubMed, the Cochrane Library, and Google Scholar were searched up to October 2024. Twenty-four studies including 139,952 patients (79,698 men and 60,254 women) were analyzed. Primary outcomes were mortality (in-hospital, 30-day, and 1-year), stroke, and treatment modality (medical vs. surgical). Secondary outcomes included complications, procedural characteristics, and hospital course. **Results:** Men were younger at diagnosis and had higher rates of substance abuse and coronary artery disease, while women more often had hypertension, diabetes, chronic lung disease, and prior valvular pathology. Men more frequently had aortic and prosthetic valve IE, whereas women had mitral and tricuspid involvement. Men were about 65% more likely to undergo surgery for infective endocarditis than women, while women were predominantly managed medically. Men had lower in-hospital (OR 0.81, 95% CI 0.72–0.92) and 1-year mortality (OR 0.76, 95% CI 0.61–0.94), though 30-day mortality did not differ significantly. Women experienced shorter hospital stays but longer ICU admissions and more heart failure, whereas men had more recurrent IE. **Conclusions:** Men underwent surgery more often and had better short- and long-term survival. Women presented later, with greater comorbidity and higher complication rates. Enhanced recognition of sex-specific risk and equitable surgical referral may improve IE outcomes.

## 1. Introduction

Infective endocarditis (IE) is a rare but highly morbid and potentially fatal disease, with a global incidence ranging from 3 to 10 per 100,000 person-years. It is characterized by infection of the endocardial surface of the heart, commonly affecting native or prosthetic valves and intracardiac devices [1]. Despite advances in diagnostic techniques and therapeutic interventions, IE continues to carry a high burden of complications, including embolic events, heart failure, stroke, and death.

Several well-recognized risk factors contribute to the development of IE, such as advanced age, poor dental hygiene, intravenous drug use, the presence of prosthetic heart valves or intracardiac devices, previous valvular disease, chronic haemodialysis, and immunosuppression [2]. However, emerging evidence suggests that biological sex itself also influences the incidence, clinical presentation, management, and outcomes of IE. These differences may be rooted in a combination of physiological, immunological, and sociocultural factors.

Physiological distinctions, such as the cardiovascular protective effects of estrogen, differences in immune system function, and variations in cardiac anatomy, have been proposed as potential contributors to sex-based differences in cardiovascular diseases, including IE [1]. Studies have also highlighted differences in the microbiological etiology of IE between men and women, with men more frequently presenting with Staphylococcus aureus or streptococcal infections, and women showing higher susceptibility to Gram-negative organisms, potentially due to higher rates of genitourinary infections [3].

Despite growing awareness of the potential impact of sex on IE, current guidelines largely lack sex-specific recommendations. Moreover, much of the existing relevant literature has been limited to single-centre studies or underpowered subgroup analyses, resulting in inconsistent and sometimes contradictory findings regarding whether sex independently affects treatment and outcomes such as mortality and embolic complications.

Sex-related disparities in medical decision-making have been increasingly scrutinized across a range of cardiovascular conditions. In the context of IE, women have been found to undergo surgical intervention less frequently than men despite having comparable or even stronger indications for surgery [4]. This discrepancy raises the possibility of implicit bias or systemic barriers affecting clinical care pathways.

Baseline sex differences in structural valve disease and cardiac anatomy may partly shape IE phenotypes and subsequent management decisions. Across valvular heart disease populations, women often present later in life and exhibit distinct remodelling patterns, including differences in ventricular geometry and, on average, smaller valve/annular dimensions compared with men. These factors may influence the distribution of affected valves, the technical feasibility of repair versus replacement, and perioperative risk assessment, and therefore are relevant when interpreting sex-based differences in surgical referral and valve-specific procedures in IE [1]. Similarly, sex differences have been described in mitral and tricuspid valve disease patterns: women may present with different distributions of degenerative versus functional regurgitation phenotypes and a higher burden of atrial functional regurgitation in older age groups, while tricuspid regurgitation is frequently under-recognized until later stages and can be influenced by right-sided pressures, atrial dilatation, and the presence of intracardiac leads that traverse the tricuspid valve.

Given the high morbidity and mortality associated with IE, and the potential for sex-based inequities in care, there is a critical need to investigate whether sex is an independent determinant of clinical trajectory in IE and whether any disparities arise from modifiable factors such as differences in treatment allocation. This systematic review and meta-analysis aims to address this knowledge gap by analyzing over 140,000 patients across 24 studies, comparing the clinical presentation, management, and outcomes of IE between men and women patients. Our objective is to determine whether biological sex is associated with differences in disease characteristics and if systemic and potentially modifiable sex-related biases play a role in observed outcome disparities.

## 2. Materials and Methods

### 2.1. Literature Search Strategy

We performed a systematic review and meta-analysis adhering to the Cochrane Collaboration published guidelines and the Preferred Reporting Items for Systematic Reviews and Meta-Analyses (PRISMA) statement. A literature search was carried out of EMBASE, MEDLINE, Cochrane, PubMed and Google Scholar from inception to October 2024. We used the search terms: (“infective endocarditis” OR endocarditis) AND (sex differences OR gender differences OR sex-based OR gender-based OR “male” OR “female” OR “men” OR “women”) AND (presentation OR clinical presentation OR symptoms OR diagnosis OR diagnostic OR management OR treatment OR therapy OR prognosis OR outcome OR mortality OR survival). Further articles were identified through use of the ‘related articles’ function on MEDLINE and a manual search of the references lists of articles found through the original search. Ethical approval was not required for this study as it is a meta-analysis of previously published data and does not involve any new collection of data from human participants Supplementary Table S1.

### 2.2. Study Inclusion and Exclusion Criteria

All comparative studies reporting sex- or gender-stratified data in patients diagnosed with IE were eligible for inclusion. We included observational designs (prospective or retrospective cohorts, registry-based analyses, and cross-sectional studies) provided they enrolled patients with a diagnosis of IE (as defined by the individual study, typically using modified Duke criteria or equivalent clinical/administrative definitions) and reported at least one prespecified outcome separately for women and men. Studies were excluded if: (1) the reported data contained inconsistencies, irreconcilable errors, or insufficient detail that precluded valid extraction of sex-stratified estimates; (2) no appropriate comparator group was available (e.g., single-sex cohorts or non-comparative series); (3) the publication type was not primary comparative research (case reports, editorials, commentaries/letters, and prior narrative or systematic reviews); or (4) the total study sample size was <10 participants, to reduce the risk of extreme small-study effects and unstable estimates. Study selection was conducted in two stages. First, one author (W.I.) independently screened titles and abstracts to identify potentially eligible records. Second, full-text articles were retrieved and assessed independently by two authors (W.I. and A.A.R.) against the predefined eligibility criteria. Any disagreements were resolved by discussion and consensus (with arbitration by a third reviewer if required).

### 2.3. Data Extraction and Critical Appraisal

Two authors (W.I. and A.AR.) read and reviewed full texts of all retrieved articles; inclusion or exclusion of studies was decided by consensus. Using a pre-established protocol, the following data were extracted: first author, study type and characteristics, number of patients, population demographics, affected valves, microbiologic profile, hospital stay duration, ICU stay duration, in-hospital mortality, 30-day mortality, 1-year mortality, haemorrhagic stroke, ischaemic stroke, overall stroke, non-stroke embolic event, acute myocardial infarction, heart failure, heart conduction abnormality, septic shock, acute kidney injury, IE recurrence, redo surgery, readmission, and followed management protocols. For this review, a data extraction sheet was developed, and pilot-tested on 3 randomly selected included studies, whereupon the sheet was refined accordingly. Data extraction was performed by one review author (W.I.). A second author (A.AR.) validated the correctness of the tabulated data. Potential inter-reviewer disagreements were resolved by consensus. Primary outcomes were overall stroke, in-hospital/30-day/1-year mortality and medical/surgical treatment. Secondary outcomes were embolic events, heart failure, conduction abnormality, septic shock, acute kidney injury, recurrence, redo surgery, readmission, hospital/ICU stay days, AV/MV/Tricuspid/Double-valve surgeries and emergency/urgent/elective surgeries.

### 2.4. Data Analysis

Odds ratios (ORs) with 95% confidence intervals (CI) and *p*-values were calculated, and Forest plots were created to represent the clinical outcomes. Chi-squared test and I2 test were executed for the assessment of statistical heterogeneity. Using a Mantel–Haenszel random-effects model, ORs were combined across the studies. Funnel plots were constructed to assess publication bias. All analyses were completed using the “metafor” package of R Statistical Software (version 4.0.2, Foundation for Statistical Computing, Vienna, Austria). A two-tailed *p* value < 0.05 was considered statistically significant.

### 2.5. Sensitivity Analysis and Publication Bias

The influence of a single study on the overall effect of patients on the main outcome was assessed by sequentially removing one study (the “leave-one-out” method). This sensitivity analysis was carried out to test the consistency of these results to investigate if individual studies had an excessive impact on the results. Publication bias was assessed through funnel plots generation.

### 2.6. Risk of Bias

Risk of bias was assessed at the study level using the ROBINS-I tool (adapted for an exposure comparison of women versus men). Two reviewers independently judged each included study across seven domains (confounding; selection of participants; classification of exposure; deviations from intended exposures; missing data; measurement of outcomes; and selection of the reported result). Domain ratings (low, moderate, or high/serious) were assigned using prespecified criteria based on study design and analytical methods and were combined to generate an overall judgement, determined by the highest level of bias in any domain. Disagreements were resolved by discussion.

## 3. Results

### 3.1. Description of Studies

**Figure 1:**
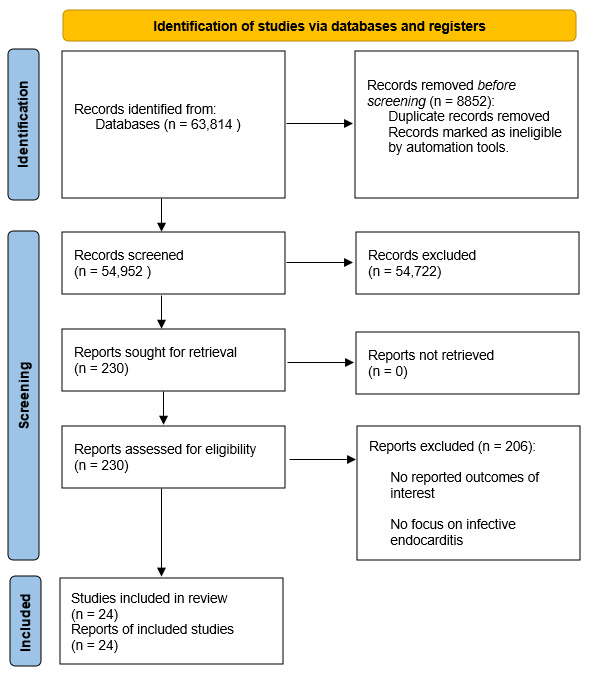
PRISMA Flow Diagram.

The literature search identified 63,814 studies, of which 54,952 were screened (Figure 1). According to the pre-established inclusion and exclusion criteria, 230 relevant articles were read in full and assessed Following critical appraisal, 24 [4,5,6,7,8,9,10,11,12,13,14,15,16,17,18,19,20,21,22,23,24,25,26,27] studies incorporating a total of 139,952 patients were included (Table 1). The studies described the management and outcomes of patients diagnosed with IE in men (79,698 patients) versus women (60,254 patients).

### 3.2. Baseline Characteristics

Baseline characteristics of the patients included in the studies are summarized in Supplementary Table S2–S7, for men and women. For continuous variables, means and standard deviations are weighted.

- Demographics

Men were younger at the time of diagnosis (mean ages 60.5 ± 10.1 years versus 62.2 ± 11.7 years, *p* = 0.01), but had a similar mean body mass index (BMI) to women (26.22 ± 1.11 kg/m2 versus 26.68 ± 1.34 kg/m2, *p* = 0.10). Men had a higher rate of tobacco use (7.97% versus 5.04%, *p* < 0.0001) and drug abuse (17.78% versus 15.07%, *p* < 0.0001), there was no significant difference in the rate of alcohol abuse (13.50% versus 8.56%, *p* = 0.785). However, women showed a higher prevalence of hypertension (51.94% versus 49.29%, *p* < 0.0001), diabetes (26.04% versus 24.46%, *p* < 0.0001), previous haemodialysis (6.82% versus 6.24%, *p* = 0.04), chronic lung disease (14.62% versus 13.91%, *p* < 0.0001) and history of valvular disease (19.09% versus 18.50%, *p* < 0.0001). In contrast, men had a wider history of coronary artery disease (22.58% versus 17.67%, *p* < 0.0001), heart failure (30.91% versus 25.51%, *p* < 0.0001), congenital heart disease (4.79% versus 3.02%, *p* = 0.01), and chronic kidney disease (24.57% versus 23.19%, *p* < 0.0001). The prevalence of previous IE in men (17.27%) and women (11.87%, *p* = 0.99) did not differ.

b. Procedures received at presentation

While men underwent more overall cardiac surgical procedures (15.42% versus 10.15%, *p* = 0.006) there was no difference in the rates of undergoing percutaneous coronary intervention (2.55% versus 1.87%, *p* = 0.99), transcatheter aortic valve implantation (54.33% versus 67.39%, *p* = 1.000), coronary artery bypass grafting (6.41% versus 3.49%, *p* = 0.99) or valvular heart surgery (7.61% versus 5.61%, *p* = 0.59). Furthermore, men more commonly received prosthetic heart valves (17.28% versus 14.53%, *p* < 0.0001) and intracardiac devices (13.98% versus 9.71%, *p* = 0.0015).

c. Valve pathology

Men had higher incidences of aortic valve endocarditis (58.76% versus 38.94%, *p* = 0.005) and prosthetic valve endocarditis (11.93% versus 7.31%, *p* = 0.0009), while a higher proportion of women had mitral valve endocarditis (55.35% versus 38.56%, *p* < 0.0001) and tricuspid/pulmonic valve endocarditis (11.73% versus 7.88%, *p* < 0.0001). There was no significant difference between men and women regarding multiple localisation endocarditis (16.98% versus 15.43%, *p* = 0.57). While vegetations were more commonly present in women than men (72.66% versus 70.56%, *p* = 0.0001), para-aortic or other abscess incidence did not differ (9.20% versus 13.63%, *p* = 0.84).

d. Microbiology

The most common etiology of IE in women was Staphylococcus aureus (25.41%), followed by other organisms (24.97%), and streptococci (17.72%). Even though men were also most affected by Staphylococcus aureus (26.24%, sex difference: *p* < 0.0001), they had a higher incidence of streptococcal IE (25.77%, sex difference: *p* < 0.001) than infection with other organisms (17.46%, sex difference: *p* < 0.0001). Enterococcal and Gram-negative IE had a higher incidence rate in men (6.38% versus 4.47%, 7.89% versus 5.60%, respectively) (sex difference: *p* < 0.0001). Culture negative endocarditis incidence did not differ between men and women (14.66% versus 16.07%, *p* = 0.057).

### 3.3. Primary Outcomes

- In-hospital Mortality

**Figure 2:**
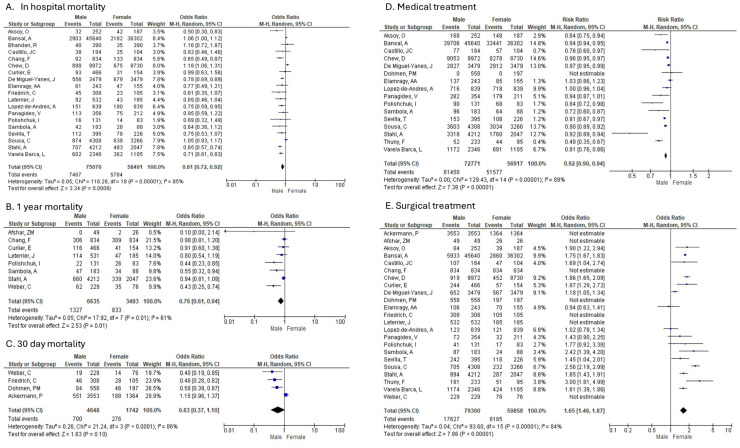
Sex-based differences in mortality and treatment approach in infective endocarditis (IE). (**A**) In-hospital mortality, (**B**) 1-year mortality, (**C**) 30-day mortality, (**D**) Medical treatment, (**E**) Surgical treatment. Forest plots display pooled odds ratios (ORs) or risk ratios (RRs) with 95% confidence intervals (CI) comparing men and women patients.

Nineteen studies compared in-hospital mortality in men and women diagnosed with IE (Figure 2A). Men had an almost 20% lower in-hospital mortality than women (random-effects model: OR: 0.81; 95% CI: 0.72 to 0.92; *p* = 0.0008). High heterogeneity was identified amongst studies reporting in-hospital mortality.

b. 30-day Mortality

Men were compared to women in 4 studies that reported on 30-day mortality in patients diagnosed with IE (Figure 2B). There was no difference in 30-day mortality (random-effects model: OR: 0.63; 95% CI: 0.37 to 1.10; *p* = 0.10). High heterogeneity was identified amongst studies reporting on 30-day mortality.

c. 1-year Mortality

Men were compared to Women in 8 studies that reported on 1-year mortality in patients diagnosed with IE (Figure 2C). Men had an almost 25% lower 1-year mortality than women (random-effects model: OR: 0.76; 95% CI: 0.61 to 0.94; *p* = 0.01). High heterogeneity was identified amongst studies reporting on 1-year mortality.

d. Overall stroke

**Figure 3:**
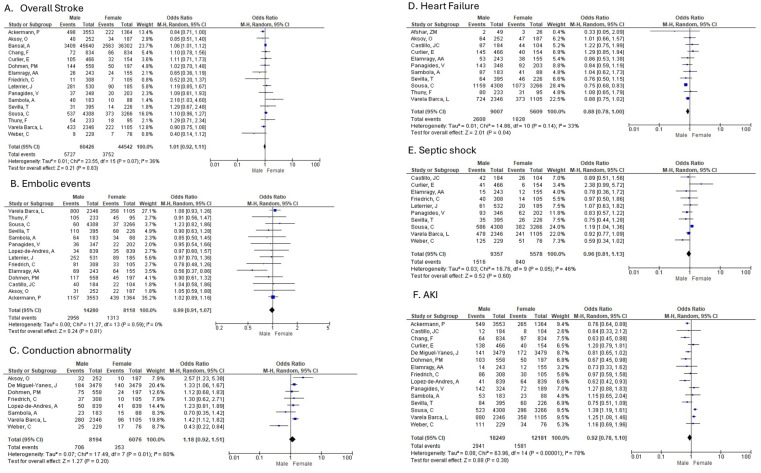
Sex-based differences in complications following infective endocarditis. (**A**) Overall stroke, (**B**) Embolic events, (**C**) Conduction abnormality, (**D**) Heart failure, (**E**) Septic shock, (**F**) Acute kidney injury (AKI). Forest plots show pooled ORs with 95% CIs for male vs. female patients.

Men were compared to Women in 16 studies that reported on overall stroke as a complication for IE (Figure 3A). There was no difference in the incidence of stroke (random-effects model: OR: 1.01; 95% CI: 0.92 to 1.11; *p* = 0.83). Low heterogeneity was identified amongst studies reporting on overall stroke.

e. Medical Treatment

Men were compared to Women in 16 studies that reported on medical treatment in patients diagnosed with IE (Figure 2D). The pooled analysis showed a statistically significant difference between sexes (random-effects model: OR: 0.92; 95% CI: 0.90–0.94; *p* < 0.00001), indicating that women were more likely to receive medical treatment alone than men. High heterogeneity was identified amongst studies reporting on medical treatment.

f. Surgical Treatment

Men were compared to Women in 23 studies that reported on surgical treatment in patients diagnosed with IE (Figure 2E). The comparison of surgical treatment between men and women showed a statistically significant difference (random-effects model: OR: 1.65; 95% CI: 1.46–1.87; *p* < 0.00001), indicating that men were more likely to undergo surgery than women. High heterogeneity was identified amongst studies reporting on surgical treatment.

### 3.4. Secondary Outcomes

- Embolic events

Men were compared to Women in 14 studies that reported on non-stroke embolic events in patients diagnosed with IE (Figure 3B). There was no statistically significant difference between men and women shown in the overall OR for non-stroke embolic events (random-effects model: OR: 0.99; 95% CI: 0.91 to 1.07; *p* = 0.81). No heterogeneity was identified amongst studies reporting on surgical treatment.

b. Heart failure

Men were compared to Women in 11 studies that reported on heart failure in patients diagnosed with IE (Figure 3D). The analysis of heart failure outcomes showed a statistically significant difference between men and women (random-effects model: OR: 0.88; 95% CI: 0.78–1.00; *p* = 0.04), with men being less likely to develop heart failure compared with women. Low heterogeneity was identified amongst studies reporting on heart failure.

c. conduction abnormality

Analysis of eight studies reporting de novo cardiac conduction abnormalities with or without intraventricular pacemaker placement in patients diagnosed with IE found no difference (random-effects model: OR: 1.18; 95% CI: 0.92 to 1.51; *p* = 0.20). Moderate heterogeneity was identified amongst studies reporting on de novo conduction abnormality.

d. septic shock

Men were compared to Women in 10 studies that reported on septic shock in patients diagnosed with IE (Figure 3E). There was no statistically significant difference when men were compared to women shown in the overall OR for septic shock (random-effects model: OR: 0.96; 95% CI: 0.81 to 1.13; *p* = 0.60). Moderate heterogeneity was identified amongst studies reporting on septic shock.

e. acute kidney injury

Men were compared to Women in 15 studies that reported on acute kidney injury (AKI) in patients diagnosed with IE (Figure 3F). There was no statistically significant difference between men and women shown in the overall OR for AKI (random-effects model: OR: 0.92; 95% CI: 0.78 to 1.10; *p* = 0.38). High heterogeneity was identified amongst studies reporting on AKI.

f. IE recurrence

**Figure 4:**
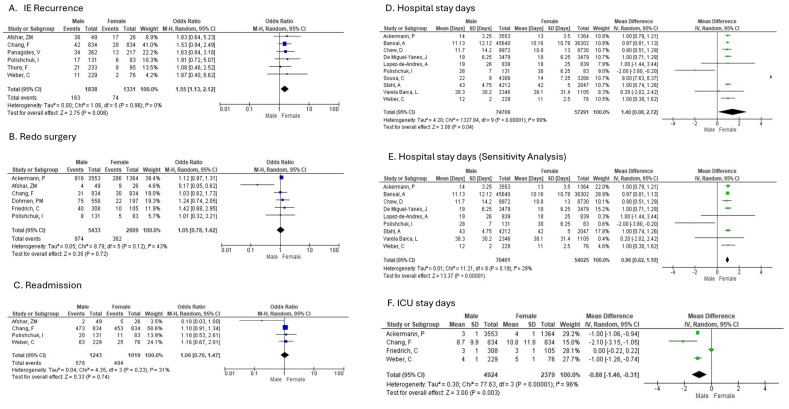
Sex-based differences in recurrence, readmission, and hospital stay in infective endocarditis. (**A**) IE recurrence, (**B**) Redo surgery, (**C**) Readmission, (**D**) Hospital stay duration, (**E**) Hospital stay duration (sensitivity analysis), (**F**) ICU stay duration. Forest plots display pooled ORs or mean differences (MD) with 95% CIs between sexes.

Men were compared to Women in 6 studies that reported on recurrence of IE (Figure 4A). The analysis of recurrence showed a statistically significant difference between men and women (random-effects model: OR: 1.55; 95% CI: 1.13–2.12; *p* = 0.006), indicating that women were 45% less likely to experience recurrence compared with men. No heterogeneity was identified amongst studies reporting on recurrence.

g. redo surgery

Men were compared to Women in 6 studies that reported on redo of surgery in patients diagnosed with IE and treated surgically (Figure 4B). There was no statistically significant difference between men and women shown in the overall OR for redo surgery (random-effects model: OR: 1.05; 95% CI: 0.78 to 1.42; *p* = 0.72). Moderate heterogeneity was identified amongst studies reporting on redo surgery.

h. Readmission

Men were compared to Women in 4 studies that reported on readmission of patients diagnosed with IE (Figure 4C). There was no statistically significant difference between men and women shown in the overall OR for readmission (random-effects model: OR: 1.06; 95% CI: 0.76 to 1.47; *p* = 0.74). Low heterogeneity was identified amongst studies reporting on redo surgery.

i. In-hospital length of stay

Ten studies compared men and women with infective endocarditis (IE) in terms of hospital length of stay (LOS) (Figure 4D). The overall analysis showed a statistically significant difference, with women having a shorter hospital stay than men (random-effects model: mean difference = 1.40 days; 95% CI: 0.08 to 2.72; *p* = 0.04). However, there was substantial heterogeneity among the studies reporting hospital stay duration.

j. ICU length of stay

Four studies compared men and women with infective endocarditis (IE) in terms of ICU length of stay (Figure 4F). The overall analysis demonstrated a statistically significant difference, with men having a shorter ICU stay than women (random-effects model: mean difference = –0.88 days; 95% CI: –1.46 to –0.31; *p* = 0.003). High heterogeneity was identified amongst studies reporting on septic shock.

k. MV surgery

**Figure 5:**
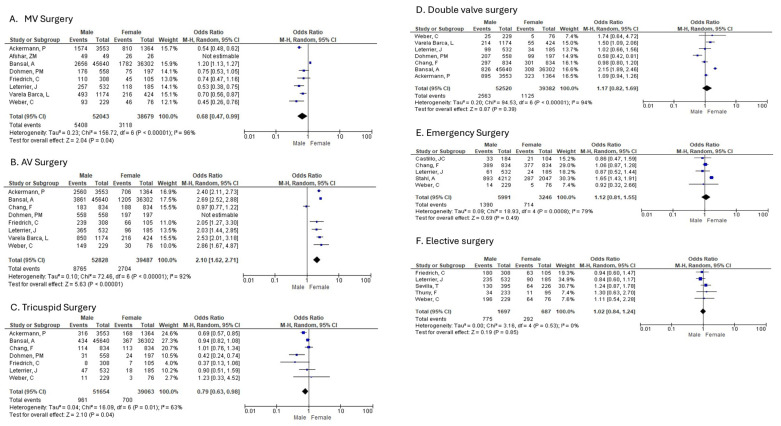
Sex-based differences in type of cardiac surgery for infective endocarditis. (**A**) Mitral valve surgery, (**B**) Aortic valve surgery, (**C**) Tricuspid valve surgery, (**D**) Double valve surgery, (**E**) Emergency surgery, (**F**) Urgent medical treatment.

Eight studies compared men and women with infective endocarditis (IE) in terms of mitral valve surgery (Figure 5A). The pooled analysis demonstrated a statistically significant difference, with women being more likely than men to undergo mitral valve surgery (random-effects model: OR = 0.68; 95% CI: 0.47 to 0.99; *p* = 0.04). Substantial heterogeneity was observed among the included studies.

l. AV surgery

Men were compared to Women in 8 studies that reported on aortic valve surgery in patients diagnosed with IE (Figure 5B). There was a statistically significant difference showing women were less likely to undergo AV surgery as compared to men (random-effects model: OR: 2.10; 95% CI: 1.62 to 2.71; *p* < 0.00001). High heterogeneity was identified amongst studies reporting on aortic valve surgery.

m. Tricuspid surgery

Men were compared to Women in 7 studies that reported on tricuspid valve surgery in patients diagnosed with IE (Figure 5C). There was a statistically significant difference showing women were more likely to undergo TV surgery (random-effects model: OR: 0.79; 95% CI: 0.63 to 0.98; *p* = 0.04). High heterogeneity was identified amongst studies reporting on tricuspid valve surgery.

n. Double-valve surgery

Men were compared to Women in 7 studies that reported on double-valve surgery in patients diagnosed with IE (Figure 5D). There was no statistically significant difference in between men and women in the overall OR for double-valve surgery (random-effects model: OR: 1.17; 95% CI: 0.82 to 1.69; *p* = 0.39). High heterogeneity was identified amongst studies reporting on double-valve surgery.

o. emergency surgery

Men were compared to Women in 5 studies that reported on emergent surgery in patients diagnosed with IE (Figure 5E). There was no statistically significant between men and women in the overall OR for emergent surgery (random-effects model: OR: 1.12; 95% CI: 0.82 to 1.55; *p* = 0.49). High heterogeneity was identified amongst studies reporting on emergent surgery.

p. urgent surgery

Men were compared to Women in 4 studies that reported on urgent surgery in patients diagnosed with IE (Supplementary Figure S1). There was a statistically significant difference between men and women shown in the overall OR of urgent surgery with women with less likely to undergo urgent surgery (random-effects model: OR: 1.43; 95% CI: 1.03 to 1.98; *p* = 0.03). Moderate heterogeneity was identified amongst studies reporting on urgent surgery.

q. elective surgery

Men were compared to Women in 4 studies that reported on elective surgery in patients diagnosed with IE (Figure 5F). There was no statistically significant difference between men and women shown in the overall OR of elective surgery (random-effects model: OR: 1.02; 95% CI: 0.84 to 1.24; *p* = 0.85). No heterogeneity was identified amongst studies reporting on elective surgery

### 3.5. Sensitivity Analysis and Publication Bias

Removing each individual study from the meta-analysis while performing the sensitivity analysis on all outcomes showed that there was no impactful change in the OR or mean difference in any of the outcomes except total in-hospital length of stay, in which heterogeneity was decreased by the removal of Sousa et al.

For total in-hospital LOS, after the removal of Sousa et al. [10] the overall mean difference demonstrated that women had a significantly lower LOS than men (random-effects model: mean difference: 0.96; 95% CI: 0.82 to 1.10; *p* < 0.0001) (Figure 4E), with low evidence of heterogeneity.

Funnel plots (Supplementary File S1) report the results of our publication bias assessment for each outcome.

### 3.6. Risk of Bias

Risk of bias was assessed using ROBINS-I (Supplementary File S1). Overall risk of bias was judged moderate for 23 of 24 studies, reflecting primarily the observational design and residual confounding. Across domains, bias due to confounding and selection of participants were the most common concerns, while classification of exposure and measurement of outcomes were generally at low risk. Only one study was rated high/serious overall, driven by greater susceptibility to confounding and selection processes.

## 4. Discussion

### 4.1. Presentation

**Figure 6:**
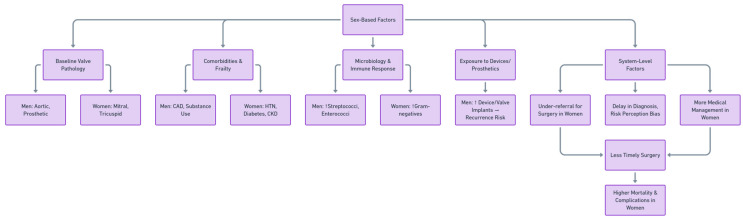
Conceptual Framework of Sex-Based Disparities in Infective Endocarditis.

This meta-analysis compares presentation, management, and outcomes of infective endocarditis between men and women (Figure 6). Consistent with existing epidemiological data, the majority of patients in our pooled analysis were men, which reflects the overall higher incidence of IE in males reported across multiple studies [28]. While the underlying explanation remains unclear, several hypotheses have been proposed: men are more frequently exposed to cardiovascular risk factors such as tobacco and drug use, and they have higher rates of pre-existing structural heart disease and intracardiac devices, all of which may predispose them to IE [2,28]. Conversely, the relative protection of premenopausal women from cardiovascular disease has been attributed to the modulatory role of estrogen, which delays disease onset and may contribute to the slightly older age at diagnosis observed in women compared with men in our analysis [29].

Our findings also revealed important differences in baseline comorbidities. Women had higher rates of hypertension, diabetes, chronic lung disease, previous haemodialysis, and prior valvular disease, while men were more likely to have coronary artery disease, heart failure, congenital heart disease, and chronic kidney disease. These sex-specific patterns align with previously described cardiovascular epidemiology, whereby women are disproportionately affected by metabolic and hypertensive comorbidities, while men present with more ischaemic and structural heart disease [28]. Such disparities likely influence both the clinical course of IE and the therapeutic decision-making process.

Regarding valve pathology, men more commonly developed aortic and prosthetic valve endocarditis, while women had a higher prevalence of mitral and tricuspid/pulmonic valve involvement. These findings mirror earlier single-centre and registry reports [11,23]. The male predominance in aortic valve disease may be explained by the higher background prevalence of bicuspid aortic valves and calcific aortic stenosis among men, both established risk factors for IE [30]. The female predominance in mitral valve IE may reflect higher baseline rates of mitral valve prolapse and rheumatic mitral pathology in women [31]. Interestingly, despite men having higher reported rates of injection drug use, women were more frequently diagnosed with tricuspid valve endocarditis, suggesting that additional mechanisms—such as sex differences in immune response or venous anatomy—may contribute [2].

Our microbiological analysis showed that Staphylococcus aureus was the leading cause of IE in both sexes, consistent with its global predominance as the primary pathogen in contemporary cohorts [2]. Men, however, demonstrated a significantly higher incidence of streptococcal, enterococcal, and Gram-negative IE. Streptococcal IE, often associated with oral and gastrointestinal portals of entry, may reflect poorer oral hygiene and higher prevalence of gastrointestinal comorbidities in men [32]. The male predominance in enterococcal and Gram-negative IE was unexpected, as women are typically more prone to genitourinary infections, a common entry point for these organisms [2]. One possible explanation is that men had higher rates of invasive cardiac procedures and prosthetic devices, which increase the risk of enterococcal and nosocomial Gram-negative IE [2]. In contrast, women had a higher proportion of “other organisms” as causative agents, which may reflect a broader spectrum of pathogens or differences in referral and diagnostic patterns. Importantly, culture-negative IE was similar between sexes, underscoring that diagnostic delays and prior antibiotic exposure affect both groups equally.

With the increasing global use of cardiac implantable electronic devices (CIEDs), device-related infections—including pocket infections and device-related infective endocarditis—have become an increasingly important clinical concern. Contemporary data indicate a steady rise in CIED infection rates, reflecting both expanding device utilization and an ageing, comorbidity-burdened patient population [33]. Management of these infections frequently necessitates complete transvenous lead extraction (TLE), which is associated with significant procedural risks and requires specialized expertise. While TLE remains the cornerstone of treatment for device-related IE, outcomes appear to differ between sexes. Recent studies have demonstrated that women undergoing TLE experience higher complication rates, worse short-term outcomes, and higher 30-day readmission rates compared with men [34]. These disparities may stem from anatomical differences, later infection recognition, smaller vessel size, or underrepresentation of women in device studies. However, across the literature, device-related and TLE-specific outcomes are often heterogeneously defined, with inconsistent reporting of sex-stratified procedural characteristics and endpoints. Consequently, a dedicated meta-analysis in this subgroup was not feasible in our study. Nevertheless, these findings collectively emphasize the need for sex-conscious procedural risk assessment, equitable referral for lead extraction, and standardized reporting of device-related IE outcomes to better characterize and mitigate sex-specific risks in this growing patient population.

### 4.2. Management

Our meta-analysis confirmed a persistent sex disparity in the management of IE. Men were significantly more likely to undergo surgical intervention than women, whereas women more frequently received medical therapy alone. This difference has been consistently reported in prior observational studies [4] and is likely multifactorial. In our pooled data, women were older at presentation and had a higher burden of comorbidities (including hypertension, diabetes, and chronic lung disease), factors that may increase perceived operative risk and bias decision-making toward conservative management. Delayed diagnosis in women, described in several studies [35], may further compound this disparity by increasing the likelihood of more advanced disease at presentation, when surgical indications may be less straightforward or operative risk is prohibitive.

Beyond overall treatment allocation, valve-specific surgical patterns were also sex-dependent. Women in our analysis were more likely to undergo mitral and tricuspid valve surgery, whereas men were more frequently treated with aortic valve surgery. These procedural patterns closely paralleled the underlying valve pathology observed in our study, with mitral and tricuspid involvement predominating in women and aortic disease predominating in men. At a mechanistic level, these differences likely reflect a combination of (i) variation in the pre-existing valvular substrate, (ii) differential exposure to healthcare-associated risk factors that shape valve involvement, and (iii) differences in referral timing and surgical candidacy.

From a pathophysiologic standpoint, the aortic valve is particularly vulnerable to infection in the presence of pre-existing structural abnormality and high-velocity turbulent flow, which promotes endothelial disruption and facilitates microbial seeding. In broader valvular disease epidemiology, men more commonly exhibit substrates that predispose to earlier or more extensive aortic pathology (including congenital or degenerative aortic valve disease), which may plausibly contribute to the predominance of aortic involvement and, consequently, a higher proportion of aortic procedures. In contrast, mitral valve involvement may be enriched in cohorts where mitral regurgitation phenotypes (degenerative or functional) are more prevalent, where left-sided filling pressures and atrial remodelling are more advanced, or where diagnosis occurs later with greater cumulative tissue destruction. These substrate differences are clinically important because they influence not only which valve becomes infected, but also the typical mode of haemodynamic decompensation (often acute severe regurgitation), the risk profile for embolisation (e.g., large, mobile vegetations), and the technical feasibility of repair versus replacement.

Tricuspid valve involvement warrants additional clinical interpretation because right-sided IE is strongly linked to healthcare-associated exposures and recurrent bloodstream inoculation, including intravascular catheters and chronic haemodialysis, and in contemporary practice may also be influenced by intracardiac hardware traversing the tricuspid valve. Even when classic risk factors such as injection drug use are not uniformly predominant, repeated vascular access and high cumulative healthcare contact can create pathways for right-sided seeding and persistent bacteraemia, which may help contextualize the higher representation of tricuspid involvement and tricuspid procedures in women reported in some cohorts.

Importantly, although women more commonly underwent mitral or tricuspid surgery, outcomes remained less favourable compared with men. Prior literature suggests that female sex is associated with lower repair rates in the setting of IE, higher reliance on prosthetic replacement, and a tendency toward bioprosthetic valve implantation, all of which may contribute to increased perioperative morbidity and mortality [1,4]. Anatomical and physiological factors may also contribute: smaller annular dimensions and higher rates of microvascular dysfunction in women can pose technical challenges and potentially worsen surgical outcomes [1,4]. Moreover, poorer outcomes despite valve-specific intervention may reflect differences in clinical state at the time of surgery—older age at diagnosis, greater comorbidity burden, delayed presentation in some series, and reduced physiological reserve—each of which can increase perioperative risk and reduce tolerance to acute valvular haemodynamic lesions. Clinically, this underscores that equalizing access to surgery is necessary but may not be sufficient on its own; earlier recognition, expedited referral, and careful optimization of comorbidities may be required to narrow outcome gaps.

Men were also more likely to receive prosthetic valves and intracardiac devices, which may reflect both a higher baseline prevalence of structural heart disease and a greater tendency toward surgical management. Prosthetic material is a recognized risk factor for recurrent IE [2], and our finding of higher recurrence in men is consistent with this association. Mechanistically, foreign material facilitates microbial adherence and biofilm formation, and recurrent or persistent infection may be more difficult to eradicate when prosthetic interfaces are involved, often prompting complex re-intervention decisions. In contrast, women demonstrated a lower likelihood of recurrence, which may relate to reduced exposure to prosthetic material and greater reliance on medical rather than surgical therapy. However, this apparent “lower recurrence” signal should be interpreted in the context of competing risks and differing management pathways, including the possibility that higher mortality or lower re-intervention rates in some cohorts could influence measured recurrence rates.

Although emergent and elective surgery did not differ significantly between sexes, men were more likely to undergo urgent surgery. This pattern suggests that men may more frequently present with acute surgical indications such as acute heart failure, abscess formation, or severe valve destruction—features that align with their higher rates of aortic valve and prosthetic valve involvement. Conversely, the higher prevalence of metabolic comorbidities in women may incline clinicians toward cautious medical optimization before surgical consideration, potentially contributing to lower overall surgical rates and longer ICU stays.

Finally, women had a shorter overall hospital stay but a longer ICU stay compared with men. A shorter hospital stay may reflect earlier transition to palliative or non-surgical pathways of care and/or higher in-hospital mortality in women. The longer ICU duration may be attributable to greater perioperative complications, delayed recovery, and more complex weaning from critical support in female patients [36].

### 4.3. Mortality

Our analysis demonstrated significant sex-based differences in mortality among patients with IE. Women experienced higher in-hospital and 1-year mortality compared with men, while no significant difference was observed at 30 days.

The observed disadvantage for women may reflect several interacting mechanisms. First, women in our study were older at presentation and carried a greater burden of comorbidities, including hypertension, diabetes, chronic lung disease, and prior valvular disease. These factors have been independently associated with poorer outcomes in IE [2] and likely increase both operative and nonoperative risks. In contrast, men more often presented with coronary artery disease and heart failure, conditions that may directly necessitate surgery and thus provide an opportunity for timely intervention.

Second, surgical treatment disparities are likely a key driver of mortality differences. Our pooled analysis confirmed that men were substantially more likely to undergo surgery, whereas women more often received medical therapy. Previous studies have consistently linked lower surgical rates in women with higher in-hospital and long-term mortality [1,4]. Importantly, surgery in IE is indicated for severe complications such as acute heart failure, uncontrolled infection, and prevention of embolization [2]. Undertreatment or delayed referral for surgery in women may therefore contribute directly to worse outcomes.

The lack of sex differences at 30-day mortality suggests that early survival is determined more by acute disease severity and procedural risks rather than sex itself. By contrast, the divergence seen at 1 year points toward the cumulative effect of treatment strategy (surgery vs. medical management), postoperative recovery, and comorbidity burden. Women’s longer ICU stays and higher complication rates, combined with lower surgical intervention, may explain their poorer long-term prognosis.

Prior reports on sex and IE mortality have been conflicting. Some studies identified female sex as an independent predictor of early mortality [13,22], while others suggested that outcomes were more strongly driven by age, comorbidities, and perioperative risk factors rather than sex per se [15]. Our findings contribute to this debate by showing that women experience a sustained mortality disadvantage, which appears to be mediated at least in part by differences in management strategy and baseline comorbidities.

### 4.4. Other Complications

Heart failure remains the most frequent complication of IE and a leading cause of death. In our pooled analysis, men were significantly less likely to develop heart failure than women. This finding contrasts with earlier reports suggesting similar rates across sexes [10]. The higher prevalence of hypertension, diabetes, and pre-existing valvular disease in women may predispose to reduced cardiac reserve and higher susceptibility to decompensation once IE develops. Furthermore, the greater burden of mitral involvement in women may also contribute, as severe mitral regurgitation is strongly associated with the development of acute heart failure in IE [2].

With regard to embolic events, no significant sex differences were observed in the incidence of overall stroke or non-stroke embolism. This is consistent with recent large-scale series showing that embolic risk in IE is determined primarily by vegetation size, mobility, and pathogen type, rather than sex alone [2]. Although women have been reported to have higher rates of thrombotic complications in other cardiovascular settings [3], our findings suggest that in IE the embolic burden is evenly distributed across sexes.

Similarly, no significant sex differences were found in conduction abnormalities or septic shock. Conduction disturbances are usually a consequence of perivalvular extension, abscess formation, or prosthetic involvement, all of which occurred at comparable rates in our analysis. The lack of difference in septic shock suggests that host inflammatory response and sepsis-related hemodynamic collapse are not strongly sex-dependent in IE.

Acute kidney injury was also comparable between men and women in our pooled analysis, in line with literature [37]. This discrepancy may reflect differences in study populations and the widespread use of nephrotoxic antibiotics across both sexes, which can overshadow any sex-specific vulnerability. Importantly, AKI remains an independent predictor of poor outcomes in IE and warrants close monitoring in all patients [2].

Our analysis demonstrated that IE recurrence was significantly more common in men. This finding may be explained by their greater exposure to prosthetic valves and intracardiac devices, both strong risk factors for recurrent infection. Higher rates of ongoing drug abuse in men may also contribute to reinfection risk [38]. In contrast, women’s lower recurrence rate may partly reflect their reduced likelihood of undergoing surgical implantation of prosthetic material.

No sex differences were observed in redo surgery or readmission rates. This suggests that once patients survive the index hospitalization and undergo surgical intervention, subsequent care pathways and risks of rehospitalization are relatively similar across sexes.

Length of stay analysis revealed important contrasts. Women had shorter overall hospital stays, but men had shorter ICU stays. A shorter hospital stay in women may reflect either higher in-hospital mortality or earlier transition to conservative/palliative pathways, both of which would shorten admission duration. Conversely, longer ICU stays in women likely reflect increased perioperative complications, delayed recovery, and greater postoperative fragility.

Valve-specific surgical outcomes further emphasized these differences. Women were more likely to undergo mitral and tricuspid valve surgery, while men more frequently underwent aortic valve surgery. These patterns reflect underlying valve pathology. However, women’s higher likelihood of urgent surgery and men’s predominance in elective and prosthetic-related interventions highlight differing clinical trajectories and decision-making paradigms.

### 4.5. Limitations

This study has several limitations. First, as a meta-analysis of observational studies, it is inherently subject to selection bias, heterogeneity in study design, and unmeasured confounding factors. Variations in diagnostic criteria, treatment protocols, and healthcare systems across included studies may have influenced the reported outcomes. Second, many studies did not adjust for potential confounders such as socioeconomic status, frailty, or timing of intervention, which could impact sex-based differences in outcomes. Third, the definition and classification of surgical urgency (emergent, urgent, elective) were not always consistent, limiting comparability. Additionally, microbiological data and reasons for surgical decision-making were variably reported, precluding more granular analysis. Despite these limitations, our study provides the most comprehensive synthesis to date of sex-related differences in infective endocarditis and highlights important disparities warranting further investigation.

Heterogeneity is an important additional limitation in this meta-analysis and in the wider IE literature. While we observed heterogeneity across several pooled outcomes, its sources are likely multifactorial and may limit the precision and generalisability of summary estimates. Potential contributors include differences in case mix (native versus prosthetic valve IE; first-episode versus recurrent disease), the proportion of healthcare-associated versus community-acquired infection, and variability in baseline valvular substrates and device prevalence across cohorts. Studies also differed in microbiological profiles (e.g., relative burdens of *Staphylococcus aureus*, streptococci, or enterococci), which can drive divergence in complication rates, embolic risk, and the need for surgery. Clinical severity at presentation and referral timing likely varied substantially between settings, including the prevalence of heart failure, abscess formation, vegetation size, and neurological events—factors that directly influence treatment selection and prognosis. In addition, heterogeneity may arise from differences in surgical candidacy thresholds, institutional access to cardiothoracic services, and multidisciplinary decision-making practices across regions and eras, as well as from variability in definitions and ascertainment of outcomes (e.g., recurrence, complications) and in follow-up duration. Finally, differential adjustment for confounding across studies—particularly for frailty, socioeconomic status, comorbidity burden, and timing of intervention—may contribute to between-study variability in observed sex differences.

## 5. Conclusions

This systematic review and meta-analysis highlights important sex-related differences in the presentation, management, and outcomes of infective endocarditis. Men were more likely to undergo surgery and had lower in-hospital and long-term mortality, whereas women presented at an older age with greater comorbidity burden, underwent fewer surgical procedures, and experienced higher complication rates. These disparities likely reflect both biological factors and gender-related differences in healthcare delivery. The underutilization of surgery in women, in particular, may represent a modifiable contributor to their poorer outcomes. Recognizing and addressing sex-specific differences through guideline adaptation, equitable surgical referral, and tailored perioperative strategies will be essential to improving care and survival for all patients with infective endocarditis.

## Figures and Tables

**Table 1:** Study characteristics.

Article	Year Published	Study Type	Timeframe	Patient Number		
				M	F	Total
Leterrier, J [5]	2024	Cohort	December 2005–December 2019	532	185	717
Chang, F [6]	2024	Retrospective Cohort	January 2000–December 2018	834	834	1668
Afshar, ZM [7]	2021	Cross-sectional	Mar 2014–October 2021	49	26	75
Aksoy, O [8]	2009	Cohort	April 1996–December 2004	252	187	439
De Miguel-Yanes, J [9]	2022	Cohort	January 2006–December 2020	3479	3479	6958
Sousa, C [10]	2021	Cross-sectional	January 2010–December 2018	4308	3266	7574
Sambola, A [11]	2010	Cohort	January 2000–December 2000	183	88	271
Bhandari, R [12]	2022	Retrospective Cohort	January 2014–December 2018	390	390	780
Weber, C [13]	2018	Retrospective Cohort	January 2009–June 2016	229	76	305
Chew, D [14]	2021	Retrospective Cohort	January 2016–December 2016	9972	8730	18,702
Curlier, E [15]	2013	Cohort	1999, 2008	466	154	620
Panagides, V [16]	2022	Retrospective Cohort	June 2005–November 2020	362	217	579
Sevilla, T [17]	2010	Retrospective Cohort	1996–2007	395	226	621
Thuny, F [18]	2012	Cohort	January 2002–December 2008	233	95	328
Polishchuk, I [19]	2020	Retrospective Cohort	2004–2013	131	83	214
Dohmen, PM [20]	2016	Retrospective Cohort	October 1994–January 2011	558	197	755
Lopez-de-Andres, A [21]	2022	Retrospective Cohort	January 2016–December 2020	839	839	1678
Friedrich, C [22]	2022	Retrospective Cohort	January 2002–February 2020	308	105	413
Varela Barca, L [23]	2021	Cohort	January 2008–December 2018	2346	1105	3451
Bansal, A [4]	2021	Retrospective Cohort	January 2004–September 2015	45,640	36,302	81,942
Castillo, JC [ 24]	2008	Retrospective Cohort	January 1987–December 2006	184	104	288
Elamragy, AA [ 25]	2020	Retrospective Cohort	February 2005–September 2016	243	155	398
Stahl, A [26]	2024	Retrospective Cohort	2010–2020	4212	2047	6259
Ackermann, P [27]	2024	Retrospective Cohort	1994–2018	3553	1364	4917

## Data Availability

The raw data supporting the conclusions of this article will be made available by the authors on request.

## Supplementary Materials

The following supporting information can be downloaded at: https://www.mdpi.com/article/10.3390/diagnostics16020260/s1, Table S1: PRISMA 2020 Checklist; Table S2: Baseline characteristics and HRBs; Table S3: Baseline comorbidities Part 1; Table S4: Baseline comorbidities Part 2; Table S5: Interventional procedures and details per gender; Table S6: Valvular pathology IE; Table S7: Microbiology Profile; Figure S1: Urgent Medical Treatment.

## References

[1] Slouha E., Al-Geizi H., Albalat B.R., Burle V.S., Clunes L.A., Kollias T.F. (2023). Sex Differences in Infective Endocarditis: A Systematic Review. Cureus.

[2] Rad A.A., Zubarevich A., Osswald A., Vardanyan R., Magouliotis D.E., Ansaripour A., Kourliouros A., Sá M.P., Rassaf T., Ruhparwar A. (2024). The surgical treatment of infective endocarditis: A comprehensive review. Diagnostics.

[3] Krcmery V., Demitrovicova A., Hricak V., Kisac P. (2010). Endocarditis due to Gram-negative bacteria. Int. J. Infect. Dis..

[4] Bansal A., Cremer P.C., Jaber W.A., Rampersad P., Menon V. (2021). Sex differences in the utilization and outcomes of cardiac valve replacement surgery for infective endocarditis: Insights from the national inpatient sample. J. Am. Heart Assoc..

[5] Leterrier J., Iung B., de Tymoski C., Deconinck L., Para M., Duval X., Provenchere S., Mesnier J., Delhomme C., Haviari S. (2024). Sex differences and outcomes in surgical infective endocarditis. Eur. J. Cardio-Thorac. Surg..

[6] Chang F.-C., Chen C.-Y., Chan Y.-H., Cheng Y.-T., Lin C.-P., Wu V.C.-C., Hung K.-C., Chu P.-H., Chou A.-H., Chen S.-W. (2024). Sex Differences in Epidemiological Distribution and Outcomes of Surgical Mitral Valve Disease. Circ. J..

[7] Afshar Z.M., Sabzi F., Shirvani M., Salehi N., Nemati N., Kheradmand W., Torbati H., Rouzbahani M. (2023). Sex-Based Differences in One-Year Outcomes After Mitral Valve Repair for Infective Endocarditis. Braz. J. Cardiovasc. Surg..

[8] Aksoy O., Meyer L.T., Cabell C.H., Kourany W.M., Pappas P.A., Sexton D.J. (2007). Gender differences in infective endocarditis: Pre- and co-morbid conditions lead to different management and outcomes in female patients. Scand. J. Infect. Dis..

[9] De Miguel-Yanes J.M., Jimenez-Garcia R., De Miguel-Diez J., Hernández-Barrera V., Carabantes-Alarcon D., Zamorano-Leon J.J., Noriega C., Lopez-De-Andres A. (2022). Differences in Sex and the Incidence and In-Hospital Mortality among People Admitted for Infective Endocarditis in Spain, 2016–2020. J. Clin. Med..

[10] Sousa C., Nogueira P.J., Pinto F.J. (2021). Gender Based Analysis of a Population Series of Patients Hospitalized with Infective Endocarditis in Portugal—How do Women and Men Compare?. Int. J. Cardiovasc. Sci..

[11] Sambola A., Fernández-Hidalgo N., Almirante B., Roca I., González-Alujas T., Serra B., Pahissa A., García-Dorado D., Tornos P. (2010). Sex differences in native-valve infective endocarditis in a single tertiary-care hospital. Am. J. Cardiol..

[12] Bhandari R., Tiwari S., Alexander T., Annie F.H., Kaleem U., Irfan A., Balla S., Wiener R.C., Cook C., Nanjundappa A. (2022). Sex Differences in Characteristics of Patients with Infective Endocarditis: A Multicenter Study. J. Clin. Med..

[13] Weber C., Gassa A., Rokohl A., Sabashnikov A., Deppe A.-C., Eghbalzadeh K., Merkle J., Hamacher S., Liakopoulos O.J., Wahlers T. (2019). Severity of Presentation, Not Sex, Increases Risk of Surgery for Infective Endocarditis. Ann. Thorac. Surg..

[14] Chew D.S., Rennert-May E., Lu S., Parkins M., Miller R.J., Somayaji R. (2021). Sex differences in health resource utilization, costs and mortality during hospitalization for infective endocarditis in the United States. Am. Heart J. Plus Cardiol. Res. Pract..

[15] Curlier E., Hoen B., Alla F., Selton-Suty C., Schubel L., Doco-Lecompte T., Minary L., Erpelding M.-L., Duval X., Chirouze C. (2014). Relationships between sex, early valve surgery and mortality in patients with left-sided infective endocarditis analysed in a population-based cohort study. Heart.

[16] Panagides V., Abdel-Wahab M., Mangner N., Durand E., Ihlemann N., Urena M., Pellegrini C., Giannini F., Scislo P., Huczek Z. (2022). Sex differences in infective endocarditis after transcatheter aortic valve replacement. Can. J. Cardiol..

[17] Sevilla T., Revilla A., López J., Vilacosta I., Sarriá C., Gómez I., García H., Román J.A.S. (2010). Influence of sex on left-sided infective endocarditis. Rev. Esp. Cardiol..

[18] Thuny F., Giorgi R., Habachi R., Ansaldi S., Le Dolley Y., Casalta J.-P., Avierinos J.-F., Riberi A., Renard S., Collart F. (2012). Excess mortality and morbidity in patients surviving infective endocarditis. Am. Heart J..

[19] Polishchuk I., Stavi V., Awesat J., Golan Y.B.B., Bartal C., Sagy I., Jotkowitz A., Barski L. (2021). Sex differences in infective endocarditis. Am. J. Med. Sci..

[20] Dohmen P.M., Binner C., Mende M., Daviewala P., Etz C.D., Borger M.A., Misfeld M., Eifert S., Mohr F.W. (2016). Gender-Based Long-Term Surgical Outcome in Patients with Active Infective Aortic Valve Endocarditis. Med. Sci. Monit..

[21] Lopez-De-Andres A., Jimenez-Garcia R., Hernández-Barrera V., De-Miguel-Díez J., De-Miguel-Yanes J.M., Martinez-Hernandez D., Carabantes-Alarcon D., Zamorano-Leon J.J., Noriega C. (2022). Sex-related disparities in the incidence and outcomes of infective endocarditis according to type 2 diabetes mellitus status in Spain, 2016–2020. Cardiovasc. Diabetol.

[22] Friedrich C., Salem M., Puehler T., Panholzer B., Herbers L., Reimers J., Hummitzsch L., Cremer J., Haneya A. (2022). Sex-Specific Risk Factors for Short- and Long-Term Outcomes after Surgery in Patients with Infective Endocarditis. J. Clin. Med..

[23] Barca L.V., Vidal-Bonnet L., Fariñas M., Muñoz P., Minero M.V., de Alarcón A., Carretero E.G., Cuadra M.G., Camacho A.M., Urkola X.K. (2021). Analysis of sex differences in the clinical presentation, management and prognosis of infective endocarditis in Spain. Heart.

[24] Castillo J.C., Anguita M.P., Delgado M., Ruiz M., Mesa D., Romo E., Crespín M., García D., Arizón J.M., de Lezo J.S. (2008). Características clínicas y pronóstico de la endocarditis infecciosa en la mujer [Clinical characteristics and prognosis of infective endocarditis in women]. Rev. Esp. Cardiol..

[25] Elamragy A.A., Meshaal M.S., El-Kholy A.A., Rizk H.H. (2020). Gender differences in clinical features and complications of infective endocarditis: 11-year experience of a single institute in Egypt. Egypt. Heart J..

[26] Stahl A., Østergaard L., Havers-Borgersen E., Strange J.E., Voldstedlund M., Køber L., Fosbøl E.L. (2024). Sex differences in infective endocarditis: A Danish nationwide study. Infection.

[27] Ackermann P., Marin-Cuartas M., Weber C., De La Cuesta M., Lichtenberg A., Petrov A., Hagl C., Aubin H., Matschke K., Diab M. (2024). Sex-related differences in patients with infective endocarditis requiring cardiac surgery: Insights from the CAMPAIGN Study Group. Eur. J. Cardiothorac. Surg..

[28] Crimmins E.M., Shim H., Zhang Y.S., Kim J.K. (2019). Differences between Men and Women in Mortality and the Health Dimensions of the Morbidity Process. Clin. Chem..

[29] Xiang D., Liu Y., Zhou S., Zhou E., Wang Y. (2021). Protective effects of estrogen on cardiovascular disease mediated by oxidative stress. Oxidative Med. Cell. Longev..

[30] Kong W.K., Bax J.J., Michelena H.I., Delgado V. (2020). Sex differences in bicuspid aortic valve disease. Prog. Cardiovasc. Dis..

[31] Stouffer G.A., Sheahan R.G., Lenihan D.J., Jacobs W., Chamoun A. (2001). Mitral valve prolapse: A review of the literature. Am. J. Med. Sci..

[32] Lipsky M.S., Su S., Crespo C.J., Hung M. (2021). Men and oral health: A review of sex and gender differences. Am. J. Men’s Health.

[33] Khalil M., Maqsood M.H., Maraey A., Elzanaty A., Saeyeldin A., Ong K., Barbhaiya C.R., Chinitz L.A., Bernstein S., Shokr M. (2023). Sex differences in outcomes of transvenous lead extraction: Insights from National Readmission Database. J. Interv. Card. Electrophysiol..

[34] Barca L., Mascia G., Di Donna P., Sartori P., Bianco D., Della Bona R., Benenati S., Merlo A.C., Buongiorno A.L., Kaufman N. (2023). Long-term outcomes of transvenous lead extraction: A comparison in patients with or without infection from the Italian region with the oldest population. J. Clin. Med..

[35] Bell A., Adegboye O.A. (2023). TThe epidemiology of infective endocarditis in New South Wales, Australia: A retrospective cross-sectional study from 2001 to 2020. Heart Lung Circ..

[36] Butterworth J., James R., Prielipp R., Cerese J., Livingston J., Burnett D. (2000). Female gender associates with increased duration of intubation and length of stay after coronary artery surgery. CABG Clinical Benchmarking Database Participants. Anesthesiology.

[37] Rodriguez Esteban M.T., Miranda Montero S., Godoy R., Quijada Fumero A., Alvarez Gonzalez L., Hernandez Afonso J. (2024). Acute renal failure in patients with infective endocarditis. Eur. Heart J..

[38] Zubarevich A., Szczechowicz M., Osswald A., Easo J., Rad A.A., Vardanyan R., Schmack B., Ruhparwar A., Zhigalov K., Weymann A. (2021). Surgical treatment of infective endocarditis in intravenous drug abusers. J. Cardiothorac. Surg..

