# Supplementary material for: Sex Disparities in Infective Endocarditis Presentation, Management and Outcomes: A Systematic Review and Meta-Analysis
Source: Diagnostics (Basel). 2026 Jan 14;16(2):260. doi: 10.3390/diagnostics16020260 (PMC12839971; doi:10.3390/diagnostics16020260)
Supplement: Supplementary file 1 [file diagnostics-16-00260-s001.zip › diagnostics-4012443-supplementary.pdf]

Article	Age				BMI				HRBs					
	M		F		M		F		Tobacco use		Alcohol Abuse		Drug Abuse	
	Mean	SD	Mean	SD	Mean	SD	Mean	SD	M	F	M	F	M	F
Leterrier, J	58.43	15.83	59.27	23.01	24.77	4.09	24.8	6.43	193	43				
Chang, F	53.7	16	53.7	17.8									32	25
Afshar, ZM	59.8	8.2	59.53	10.25	26.35	4.51	27.84	5.13	19	2				
Aksoy, O													18	7
De Miguel-Yanes, J	70.11	11.87	70.25	17.59									60	41
Sousa, C	69	32.63	76	26.7										
Sambola, A	58	18	63	16										
Bhandari, R	41.42	39.99	34.94	28.3					299	312	144	79	250	303
Weber, C	61.9	14.4	63.1	16.3	26.4	4.8	26.8	7.2	58	10	25	2	10	7
Chew, D	60.8	18.3	61.4	21									1548	1569
Curlier, E	60.4	14.5	64.3	15.9					119	21			15	3
Panagides, V	77.9	7.5	80	7.5	28	5.7	27.9	6.1						
Sevilla, T	59	15	63	15										
Thuny, F	61	15	62	18										
Polishchuk, I	58	18.9	62.4	16.4									19	5
Dohmen, PM	61.6	13.7	64.9	14.1										
Lopez-de-Andres, A	76.29	7.65	75.58	9										
Friedrich, C	62.33	17.13	65.67	13.53	25.9	4.47	26.63	5.86	78	25			16	7
Varela Barca, L	64.5	14.4	68.4	14.9					405	77				

Bansal, A	59.85	17.85	59.85	19.62					2811	1441			9004	5641
Castillo, JC	50	18	52	21										
Elamragy, AA	31.67	11.93	29.33	10.48									40	2
Stahl, A	70.77	12.75	73.83	13.28							483	134		
Ackermann, P	63	14.09	67	13.36	25.87	3.63	26.13	5.42						

Supplementary Table S2: Baseline characteristics and HRBs

Article	Medical History															
	HTN		DM		CAD		HF		CKD		Dialysis		AV Disease		MV Disease	
	M	F	M	F	M	F	M	F	M	F	M	F	M	F	M	F
Leterrier, J	231	73	86	32	50	7	122	50			21	14				
Chang, F	316	327	176	184	160	158	186	186	211	192	72	71				
Afshar, ZM	24	21	10	15												
Aksoy, O			64	76							45	54				
De Miguel-Yanes, J			793	816	379	343	382	392	656	640			789	848	1069	1080
Sousa, C	1527	1301	1116	900	606	364			515	372	192	132	611	292	564	349
Sambola, A			32	18							6	9				
Bhandari, R	132	98	65	58	62	33			45	25						
Weber, C	152	48	63	25	64	23	229	76			15	11				
Chew, D	5941	5062	3090	2580			2247	2012	3070	2391						
Curlier, E	171	70	83	38							9	8				
Panagides, V			145	71					150	98			362	217	84	58
Sevilla, T			67	59												
Thuny, F			31	12												
Polishchuk, I			34	23	41	19	23	9	10	7						
Dohmen, PM			159	72			318	116	113	113	31	14				

Lopez-de-Andres, A			839	839	132	114	133	146	247	257			192	206	260	268
Friedrich, C	177	63	90	41	142	36	65	18	92	24	11	7	45	24	4	2
Varela Barca, L	1229	679	637	329					600	264						
Bansal, A	21825	18662	10068	8618	10502	6755										
Castillo, JC																
Elamragy, AA			13	7					28	19						
Stahl, A	2416	1217	921	431	1465	516	1012	415	679	335						
Ackermann, P	1764	728	867	418	951	286	2901	1156	1331	553	298	120				

Supplementary Table S3: Baseline comorbidities Part 1

	Overall Valvular Disease		Congenital Heart D		Afib		Liver Disease		Chronic Lung Disease		HIV		Neoplasia / Cancer		Immunosuppression		Previous IE	
	M	F	M	F	M	F	M	F	M	F	M	F	M	F	M	F	M	F
Leterrier, J					61	27	26	9	49	8			60	27			140	47
Chang, F							24	24	51	51								
Afshar, ZM																		
Aksoy, O			31	16							8	6	34	25	24	31		
De Miguel-Yanes, J			87	105	1223	1167			131	104								
Sousa, C			24	18			279	87	397	305	107	26	627	391				
Sambola, A											19	1	26	12	6	12		
Bhandari, R									45	44								
Weber, C			5	5					25	4	4	1	31	10	1	3	12	8
Chew, D	4044	4168					1081	734	2104	2145								
Curlier, E	226	74											62	24	29	10		
Panagides, V	362	217			148	99			111	47							2	4
Sevilla, T																		
Thuny, F																		
Polishchuk, I							16	4	8	13			13	10				
Dohmen, PM									68	15			106	37				
Lopez-de-Andres, A	526	570			309	319			40	35								
Friedrich, C	112	42					42	13	37	13			40	15	6	5	51	9

Varela Barca, L							130	25	482	135			400	164				
Bansal, A	3194	1931	2382	1147	9643	7405	1711	849					1916	1339				
Castillo, JC			52	18													12	7
Elamragy, AA			24	8			14	0					0	7			12	3
Stahl, A					1195	566	138	72	517	311			872	472				
Ackermann, P									394	121								

Supplementary Table S4: Baseline comorbidities Part 2

Article	Interventional History													
	PCI		TAVI		CABG		Valvular Heart Surgery		Overall Heart Surgery		Prosthetic Valve Carrier		Devices	
	M	F	M	F	M	F	M	F	M	F	M	F	M	F
Leterrier, J	24	7			4	1	164	50	168	51				
Chang, F														
Afshar, ZM														
Aksoy, O													36	14
De Miguel-Yanes, J											344	322		
Sousa, C	79	29			136	47			136	47	537	379	386	263
Sambola, A														
Bhandari, R														
Weber, C	11	4			18	3	46	23	64	26	48	21		
Chew, D														
Curlier, E											95	32		
Panagides, V			362	217					101	28	362	217		
Sevilla, T											145	105		
Thuny, F														
Polishchuk, I													18	12
Dohmen, PM														
Lopez-de-Andres, A											84	83		
Friedrich, C			2	0	7	2	112	42	125	46				
Varela Barca, L									825	427				
Bansal, A	1177	704			3071	1335	3194	1931	6265	3266				
Castillo, JC														
Elamragy, AA														

Stahl, A											854	338	805	253
Ackermann, P									1066	417				

Supplementary table S5: Interventional procedures and details per gender

Article	Affected Valves																					
	AV Reg		AV IE		MV Reg		MV IE		TV / PV Reg		TV / PV IE		Prosthetic IE		Multiple Localisation IE		Vegetations		Intracardiac Abcesses		Overall Abcesses	
	M	F	M	F	M	F	M	F	M	F	M	F	M	F	M	F	M	F	N	N	N	N
Leterrier, J	219	56			56	63															208	62
Chang, F							834	834														
Afshar, ZM					49	26			49	26												
Aksoy, O													51	32			161	130	45	18	45	18
De Miguel-Yanes, J																						
Sousa, C																					106	58
Sambola, A	63	20	85	27	45	30	65	46			33	15					160	77	20	8	20	8
Bhandari, R			139	60			111	124			152	223										
Weber, C			106	23			77	40			8	3	9	4			181	65	81	32	81	32
Chew, D																						
Curlier, E			204	54			165	77					95	32	82	19	428	139	106	23	106	23
Panagides, V			194	90			48	38			19	13			101	76						
Sevilla, T			172	49			138	95					115	105							57	28
Thuny, F			153	35													185	80			74	24

Polishchuk, I	4	3	35	26	34	18	38	30	22	14	18	4			10	4	63	35				
Dohmen, PM	482	159	558	197			124	54					157	53	133	60	318	112			64	23
Lopez-de-Andres, A																						
Friedrich, C	87	21	107	21	55	23	61	31	3	5	4	3	104	39			209	76	81	32	81	32
Varela Barca, L			1158	358			693	538			125	49	752	383	330	112	1874	880	478	198	478	198
Bansal, A													4116	1818								
Castillo, JC			92	30			73	56					51	37			134	73			22	12
Elamragy, AA													69	47			184	114	32	20	53	29
Stahl, A																						
Ackermann, P			2484	667			1405	773			232	92	944	373			2232	917				

Supplementary Table S6: Valvular pathology IE

Article	Microbiologic Profile																	
	Oral Streptococcus		S. Bovis		Streptococci		S. Aureus		Staphylococci		Enterococcus		G- Bacteria		Other		Culture (-)	
	M	F	M	F	M	F	M	F	M	F	M	F	M	F	M	F	M	F
Leterrier, J	95	32	49	13	63	33	132	46	22	10	51	12	45	19	28	6		
Chang, F																		
Afshar, ZM																		
Aksoy, O	29	16					91	76	39	17	26	19			38	33	29	26
De Miguel-Yanes, J																		
Sousa, C					728	302	308	162	812	430	359	176	453	445	30	9		
Sambola, A	43	19					51	23	12	7	17	11			52	25	8	3
Bhandari, R	21	5			43	26	239	297			42	18			91	90	16	16
Weber, C							56	34			33	4			90	27	50	11
Chew, D																		
Curlier, E	95	31	107	20	30	20	95	35	26	12	48	12			34	9		
Panagides, V	50	21	19	7	15	7	81	56	65	32	91	52					15	20
Sevilla, T	66	24	16	9			71	44	63	40	38	13	10	19	7	7	63	31
Thuny, F					101	34			56	24	35	14			41	23		
Polishchuk, I	15	7	6	6	5	6	33	17	19	3	16	10	6	5	17	6	11	19
Dohmen, PM																		

Lopez-de-Andres, A																		
Friedrich, C	35	8			26	11	69	27	18	10	48	13			4	3	85	28
Varela Barca, L					697	292	507	311	403	187	407	139						
Bansal, A					12624	6530	12742	9453			1839	1154	3509	1757	9319	10480		
Castillo, JC	39	23					37	27	25	14	27	13			35	10	18	16
Elamragy, AA									69	33							168	106
Stahl, A					1166	579	1213	708	273	139	762	216			248	88	547	315
Ackermann, P					603	190			853	407	424	117						

Supplementary Table S7: Microbiology Profile

A Urgent medical

Study or Subgroup	Male Events	Male Total	Female Events	Female Total	Weight	Odds Ratio M-H, Random, 95% CI
Leterrier, J	235	532	70	185	33.8%	1.30 [0.92, 1.83]
Sevilla, T	112	395	54	226	31.4%	1.26 [0.87, 1.84]
Thuny, F	147	233	40	95	24.5%	2.35 [1.44, 3.82]
Weber, C	19	229	7	76	10.3%	0.89 [0.36, 2.21]
**Total (95% CI)**		**1389**		**582**	**100.0%**	**1.43 [1.03, 1.98]**
Total events	513		171			

Heterogeneity: $Tau^2 = 0.05$; $Chi^2 = 5.77$, df = 3 (P = 0.12); $I^2 = 48\%$
Test for overall effect: Z = 2.17 (P = 0.03)

Odds Ratio
M-H, Random, 95% CI

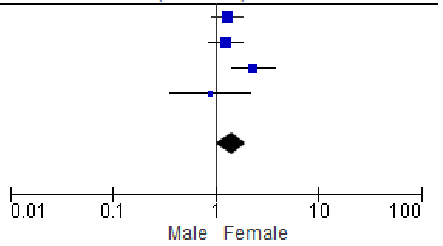

Supplementary figure S1: Urgent Medical Treatment

In hospital mortality

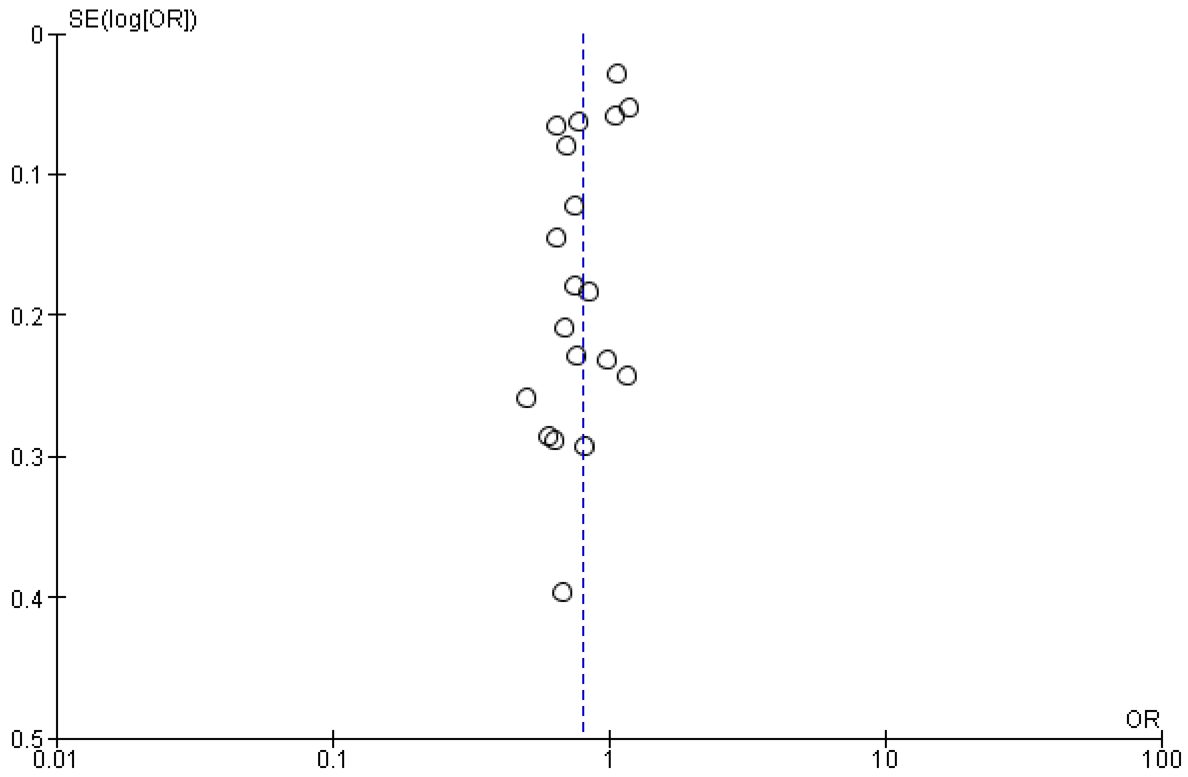

Stroke

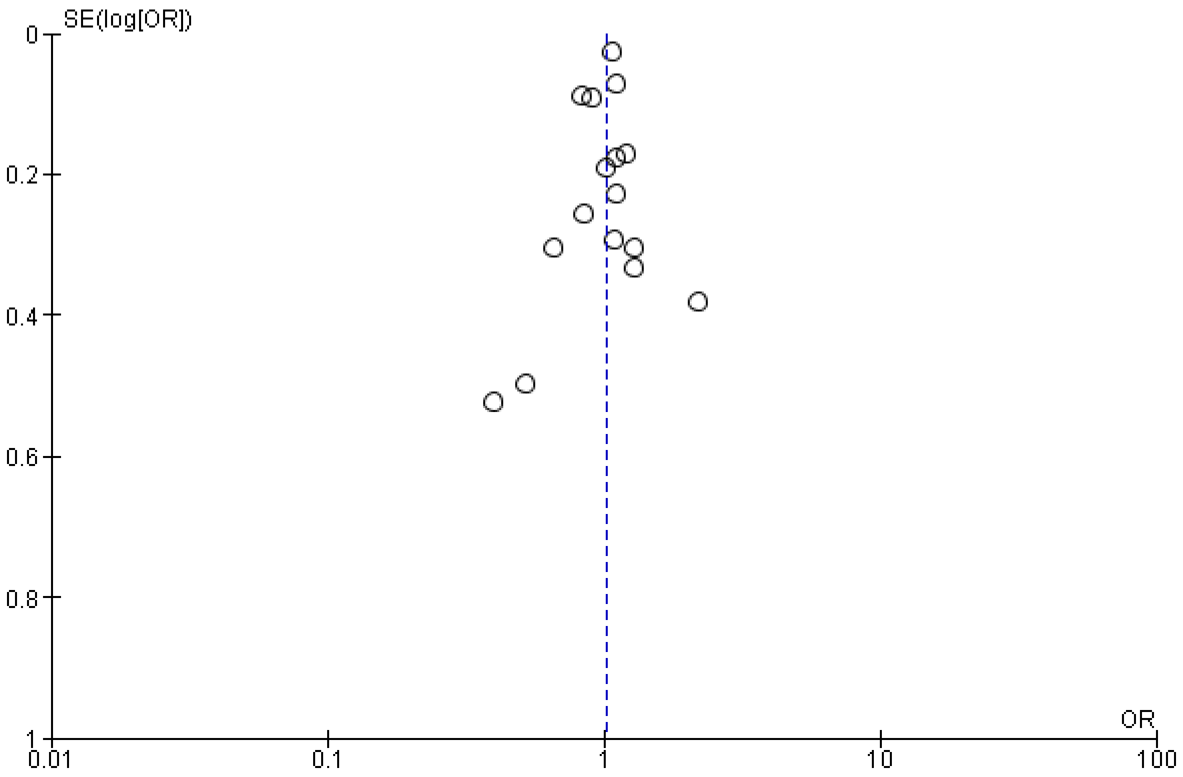

Embolism

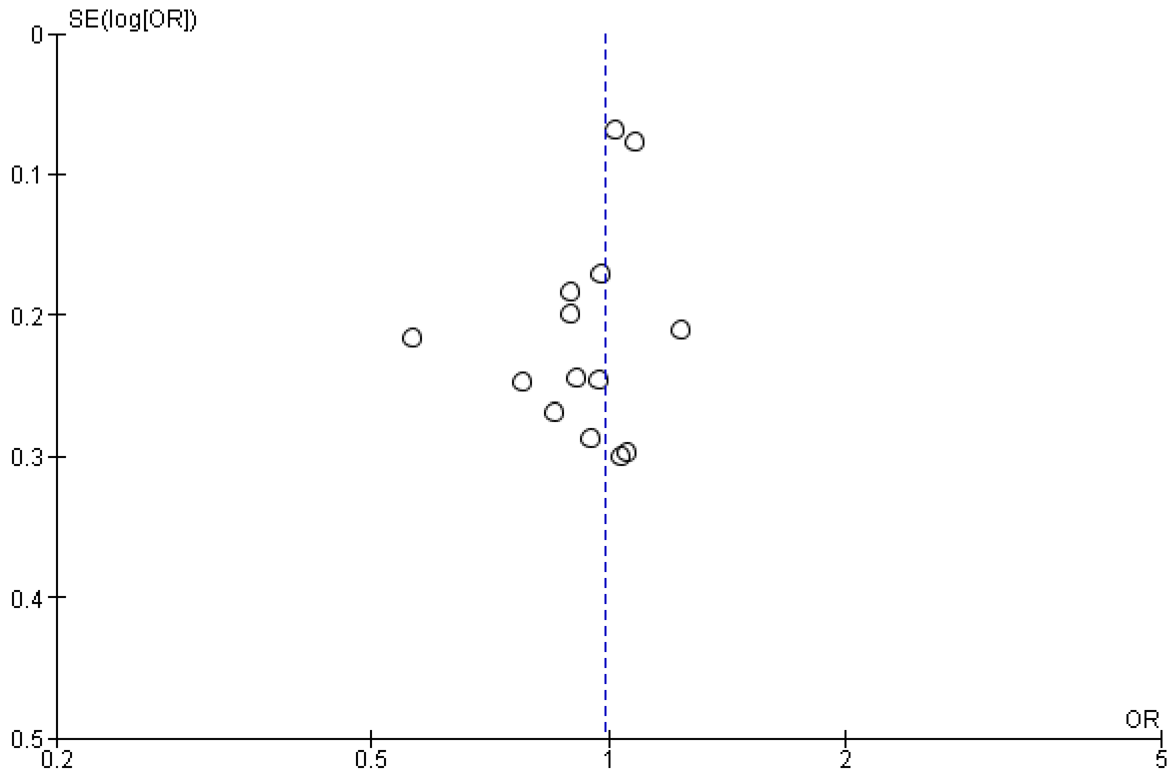

Heart failure

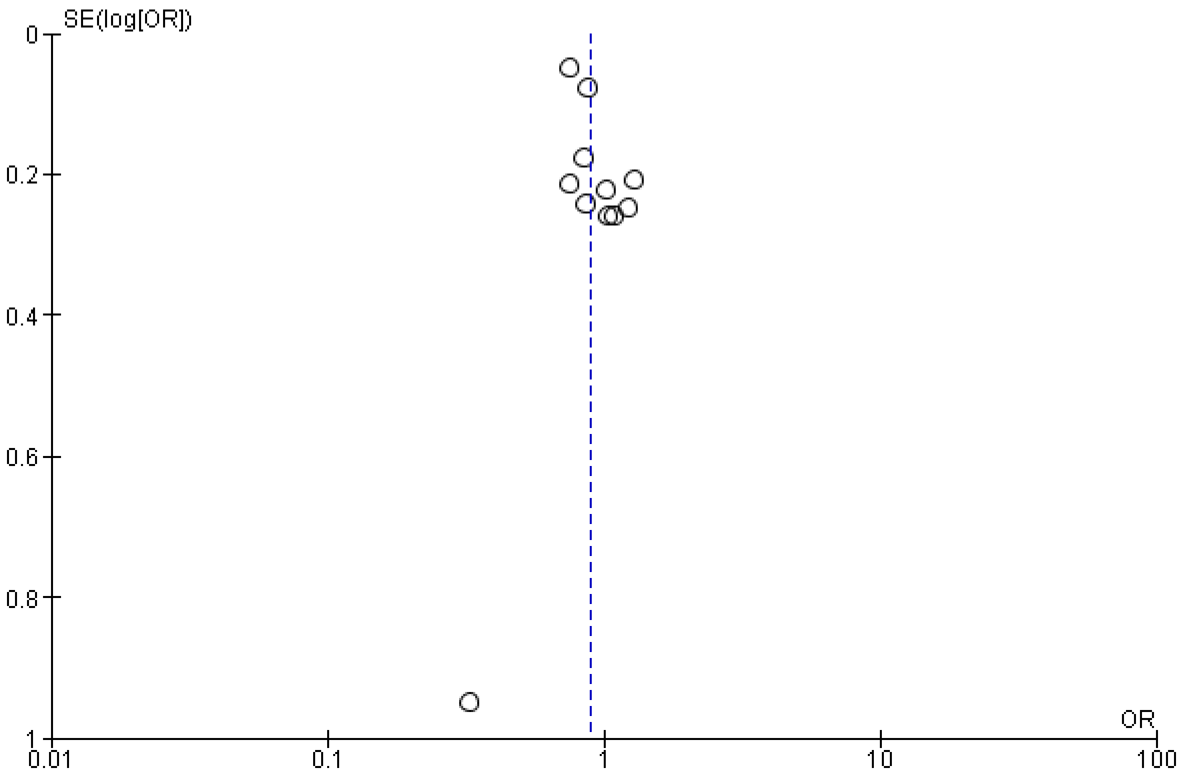

Follow up mortality

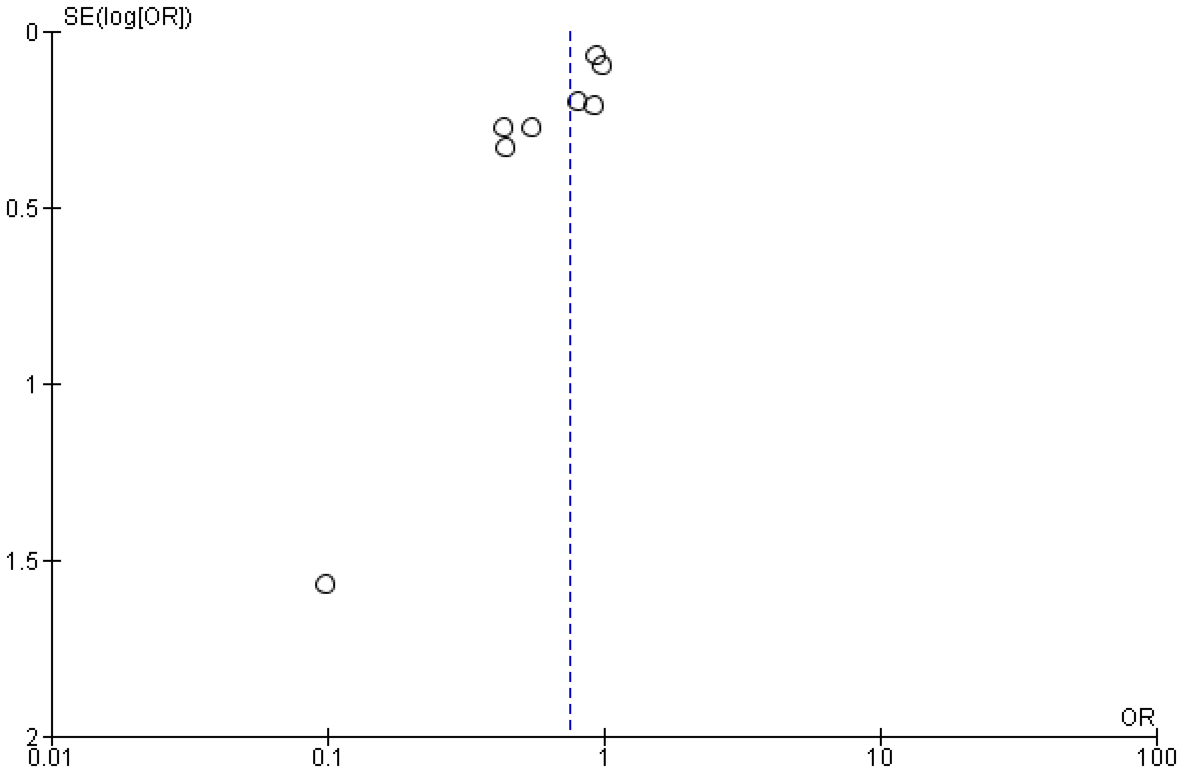

Conduction abnormality

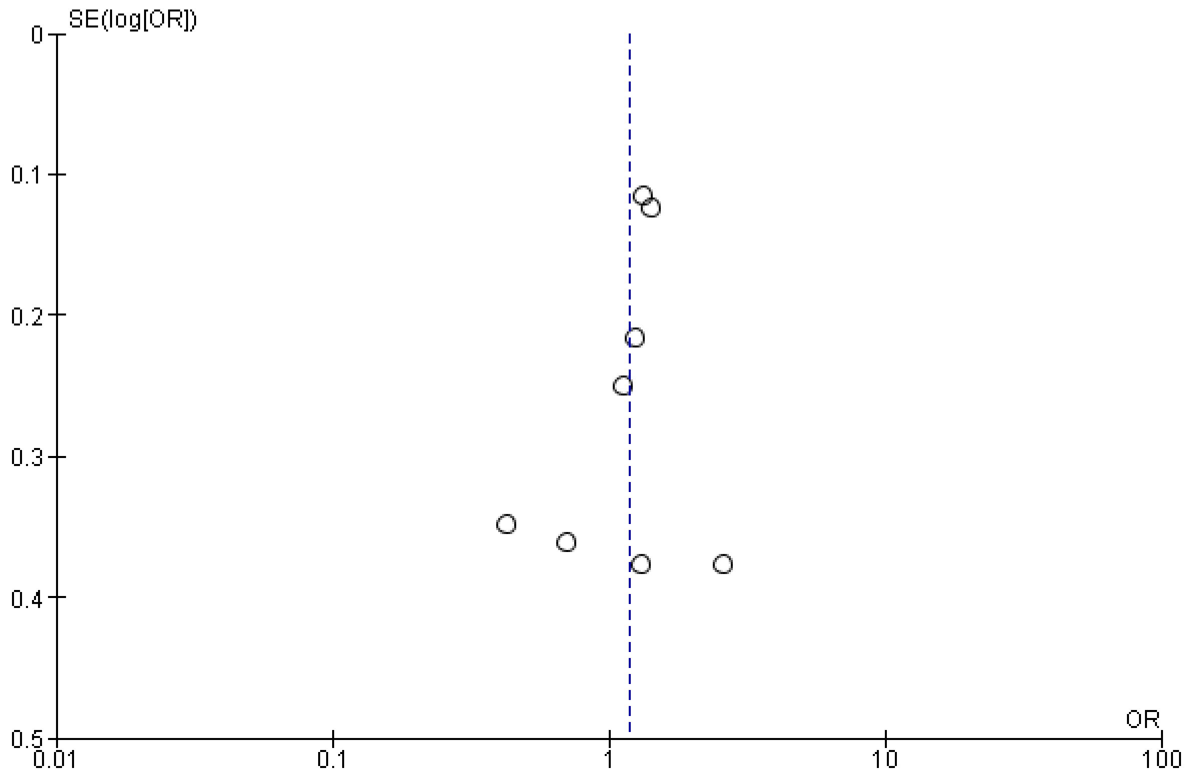

Septic shock

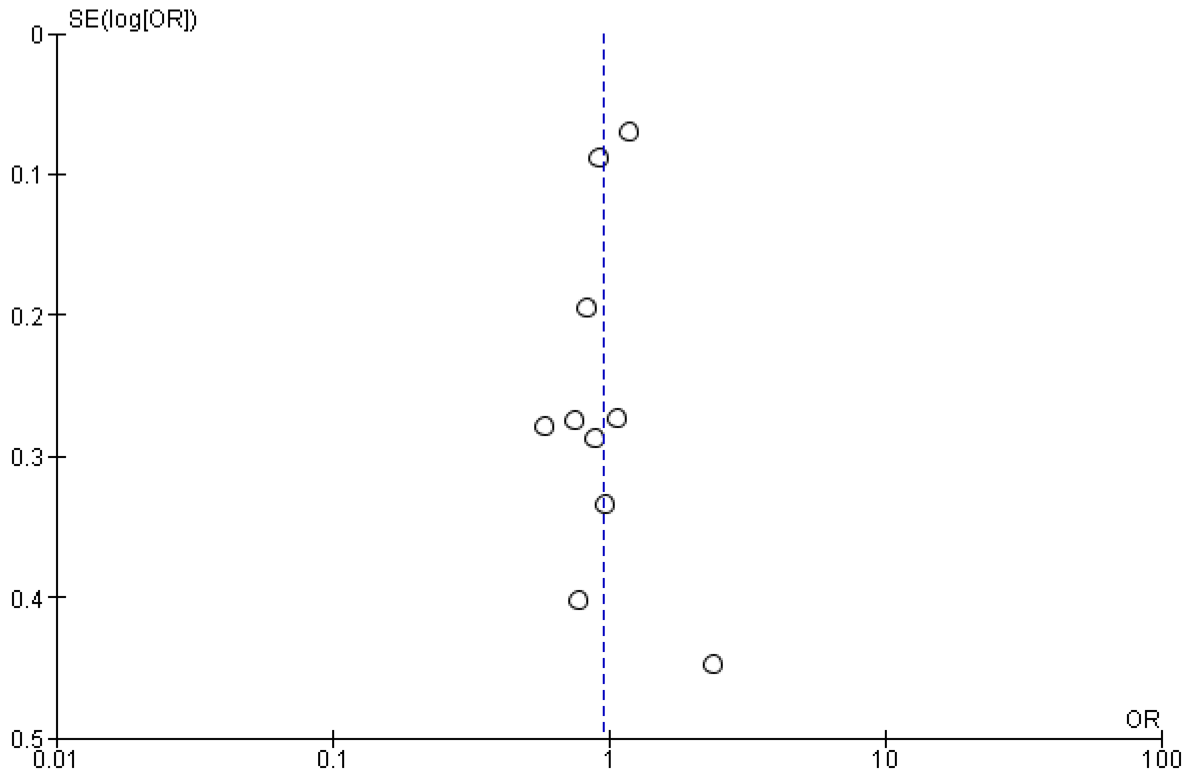

AKI

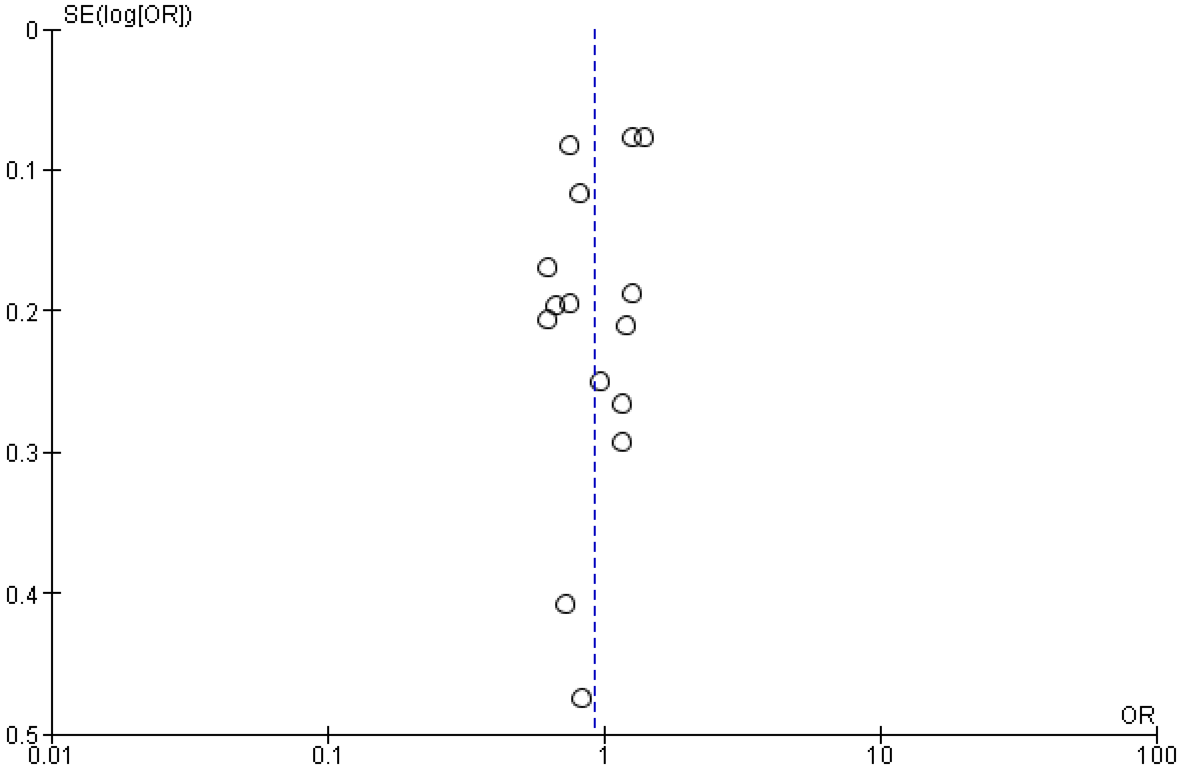

IE recurrence

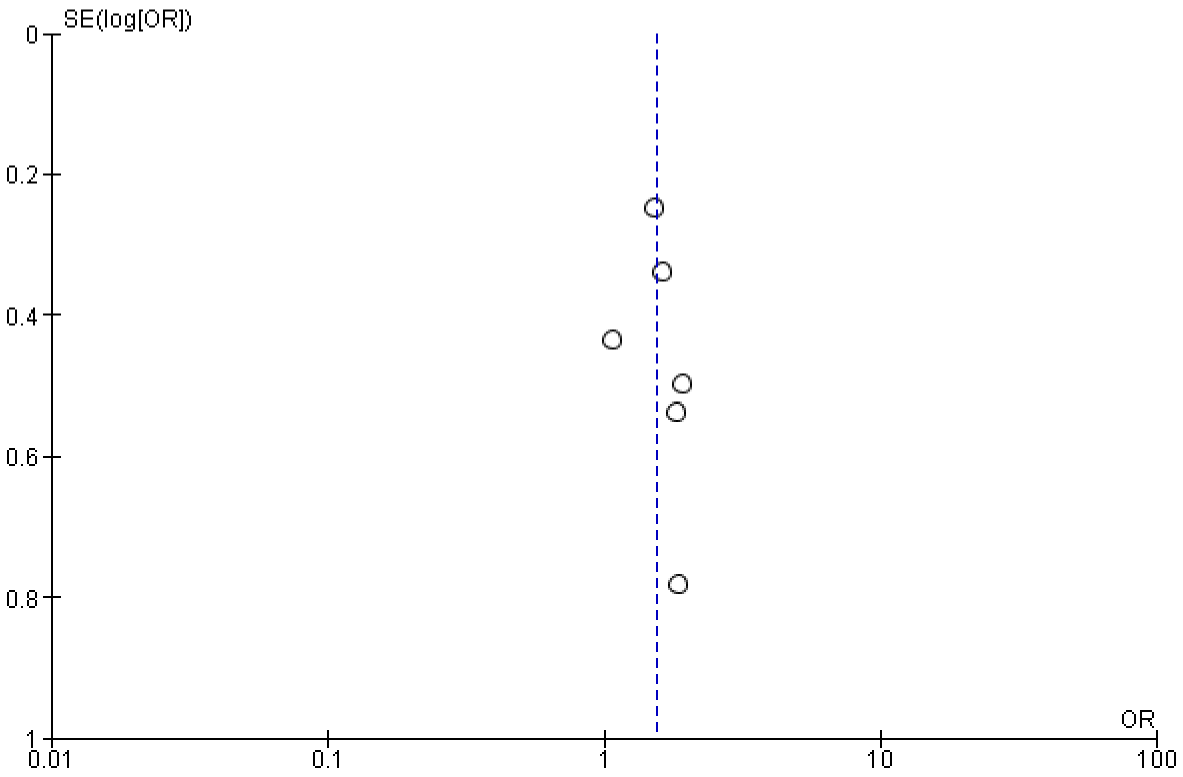

Redo surgery

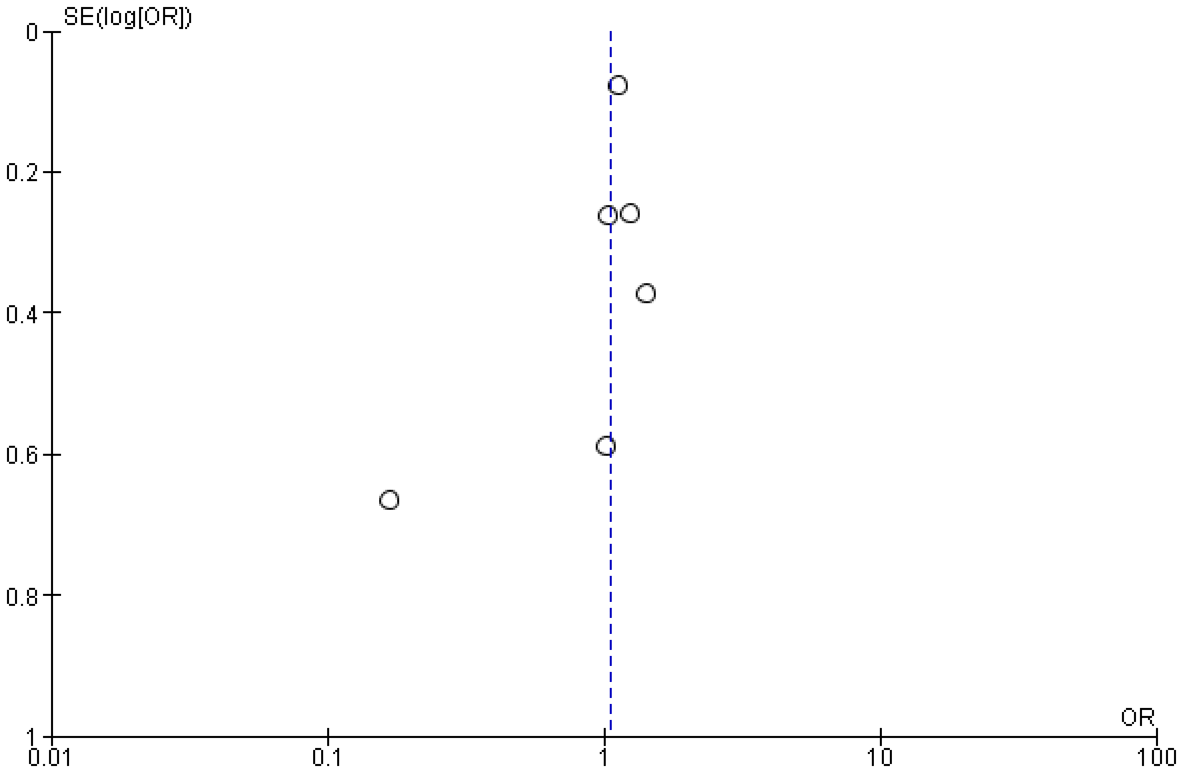

Readmission

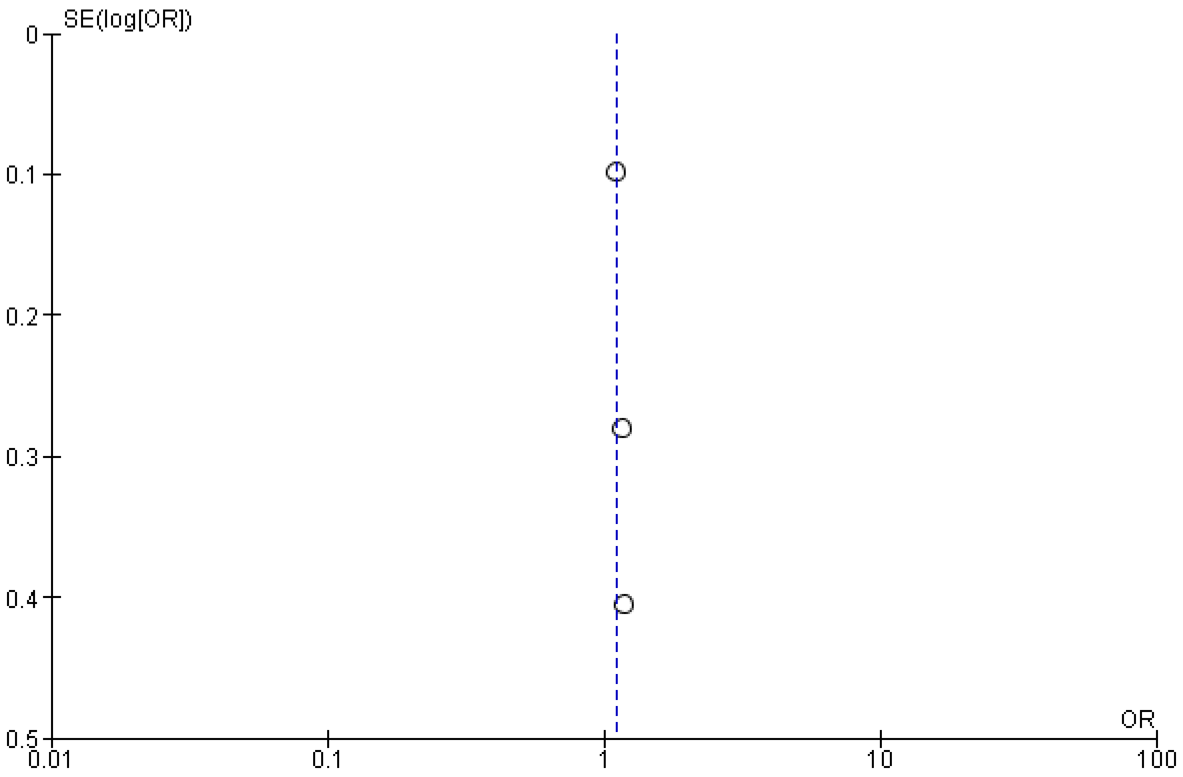

30 day mortality

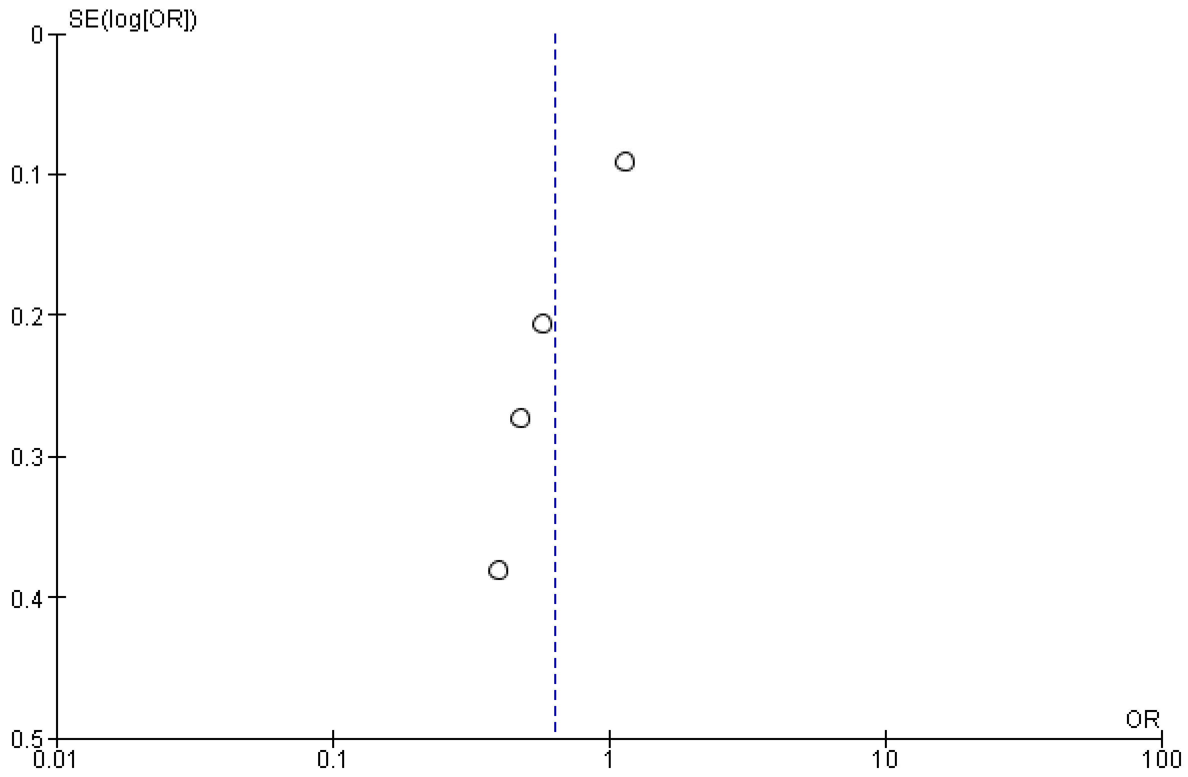

Hospital Stay

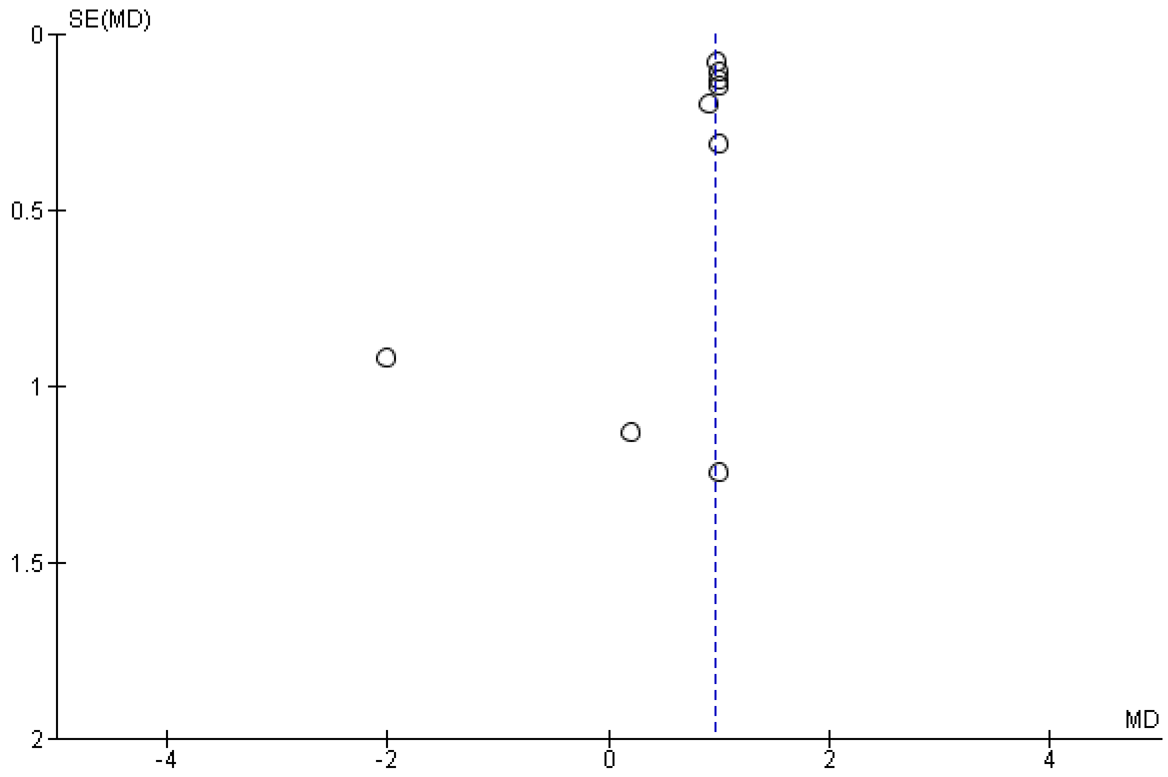

ICU Stay

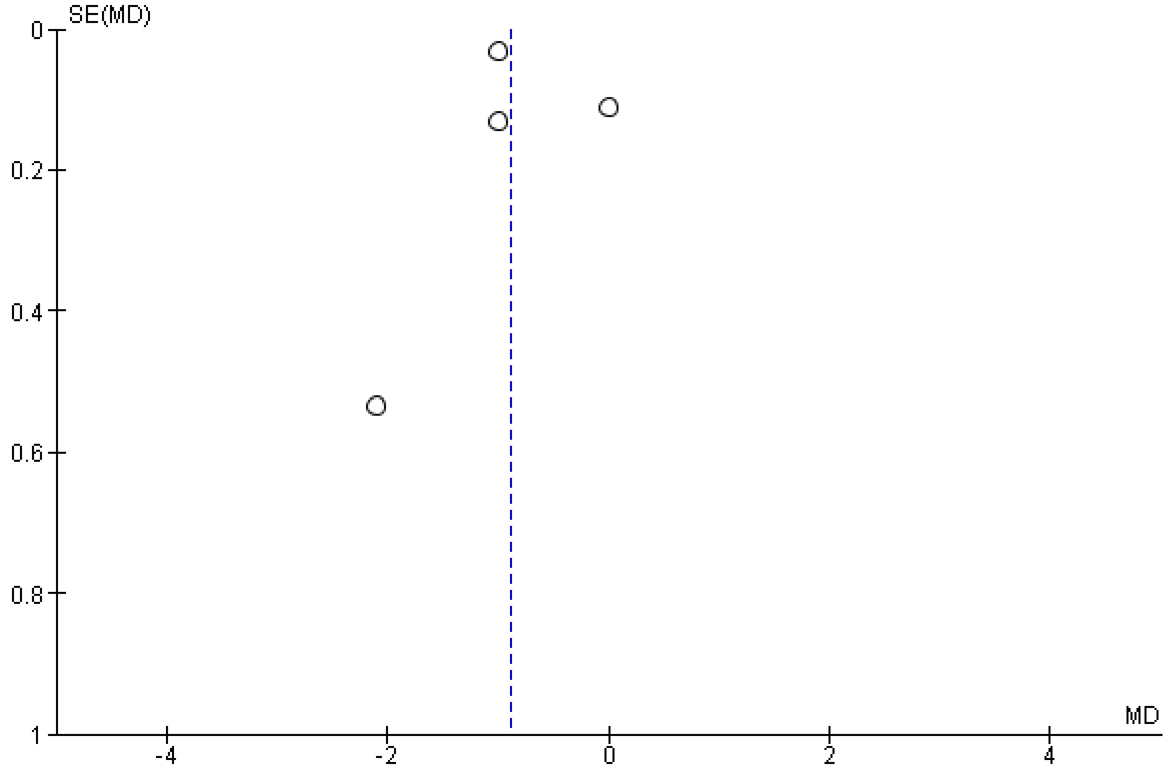

Medical treatment

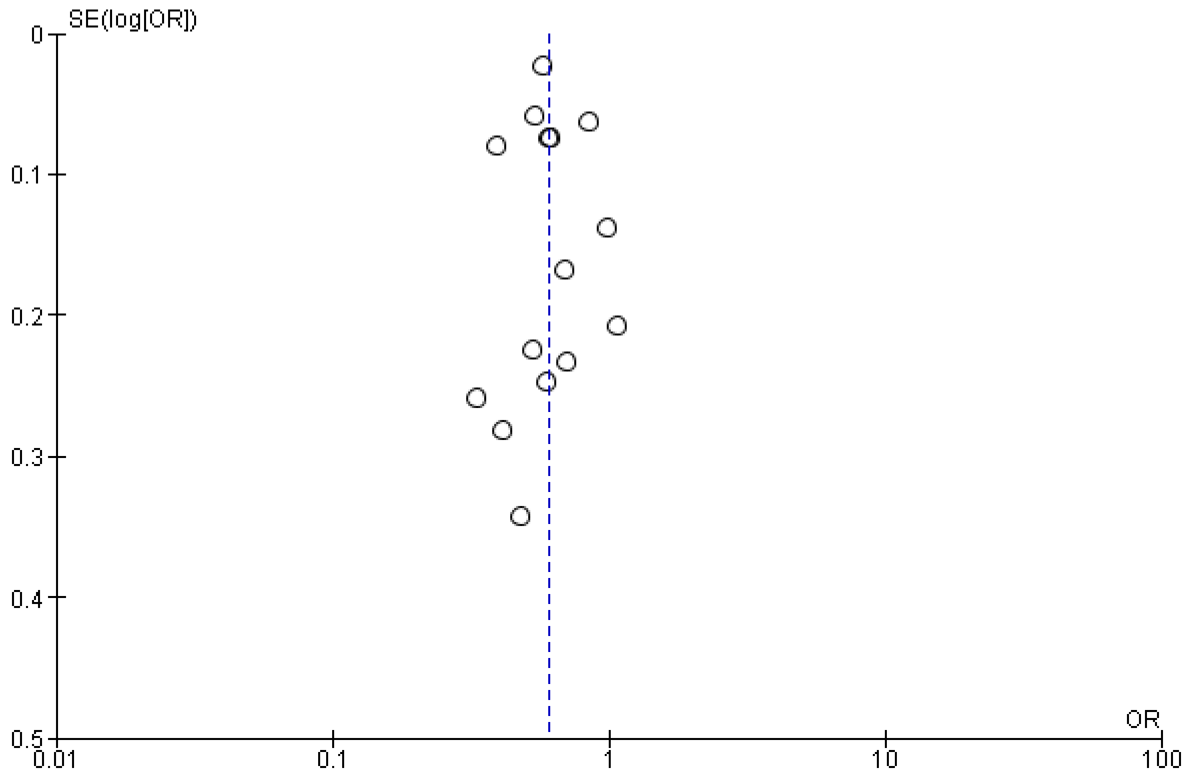
SE(log[OR])
0
0.1
0.2
0.3
0.4
0.5
OR
0.01
0.1
1
10
100

Study	Bias due to confounding	Bias in selection of participants	Bias in classification of interventions	Bias due to deviations from intended interventions	Bias due to missing data	Bias in measurement of outcomes	Bias in selection of the reported result	Overall
Leterrier, J	Moderate	Moderate	Low	Low	Moderate	Low	Moderate	Moderate
Chang, F	Moderate	Moderate	Low	Low	Moderate	Low	Moderate	Moderate
Afshar, ZM	Serious	Moderate	Low	Low	Moderate	Low	Moderate	Serious
Aksoy, O	Moderate	Moderate	Low	Low	Moderate	Low	Moderate	Moderate
De Miguel-Yanes, J	Moderate	Low	Low	Low	Low	Moderate	Moderate	Moderate
Sousa, C	Moderate	Low	Low	Low	Low	Moderate	Moderate	Moderate
Sambola, A	Moderate	Moderate	Low	Low	Moderate	Low	Moderate	Moderate
Bhandari, R	Moderate	Moderate	Low	Low	Moderate	Low	Moderate	Moderate
Weber, C	Moderate	Moderate	Low	Low	Moderate	Low	Moderate	Moderate
Chew, D	Moderate	Low	Low	Low	Low	Moderate	Moderate	Moderate
Curlier, E	Moderate	Low	Low	Low	Low	Moderate	Moderate	Moderate
Panagides, V	Moderate	Moderate	Low	Low	Low	Low	Moderate	Moderate
Sevilla, T	Moderate	Moderate	Low	Low	Moderate	Low	Moderate	Moderate
Thuny, F	Moderate	Moderate	Low	Low	Moderate	Low	Moderate	Moderate
Polishchuk, I	Moderate	Moderate	Low	Low	Moderate	Low	Moderate	Moderate
Dohmen, PM	Moderate	Moderate	Low	Low	Moderate	Low	Moderate	Moderate
Lopez-de-Andres, A	Moderate	Low	Low	Low	Low	Moderate	Moderate	Moderate
Friedrich, C	Moderate	Moderate	Low	Low	Moderate	Low	Moderate	Moderate
Varela Barca, L	Moderate	Low	Low	Low	Low	Moderate	Moderate	Moderate
Bansal, A	Moderate	Moderate	Low	Low	Low	Moderate	Moderate	Moderate
Castillo, JC	Moderate	Moderate	Low	Low	Moderate	Low	Moderate	Moderate
Elamragy, AA	Moderate	Moderate	Low	Low	Moderate	Low	Moderate	Moderate

Stahl, A	Moderate	Low	Low	Low	Low	Moderate	Moderate	Moderate
Ackermann, P	Moderate	Moderate	Low	Low	Moderate	Low	Moderate	Moderate
